# Solid-Phase Synthesis of 2-Benzothiazolyl and 2-(Aminophenyl)benzothiazolyl Amino Acids and Peptides

**DOI:** 10.3390/molecules28145412

**Published:** 2023-07-14

**Authors:** Spyridon Mourtas, Vasileios Athanasopoulos, Dimitrios Gatos, Kleomenis Barlos

**Affiliations:** 1 Department of Chemistry, University of Patras, 26510 Rio Patras, Greece; up1061182@upnet.gr (V.A.); d.gatos@upatras.gr (D.G.); 2 CBL-Patras, Patras Industrial Area, Block 1, 25018 Patras, Greece

**Keywords:** solid-phase synthesis, 2-benzothiazoles, 2-(aminophenyl)benzothiazoles, amino acids, racemization, chiral purity

## Abstract

2-benzothiazoles and 2-(aminophenyl)benzothiazoles represent biologically interesting heterocycles with high pharmacological activity. The combination of these heterocycles with amino acids and peptides is of special interest, as such structures combine the advantages of amino acids and peptides with the advantages of the 2-benzothiazolyl and 2-(aminophenyl)benzothiazolyl pharmacophore group. In this work, we developed an easy and efficient method for the solid-phase synthesis of 2-benzothiazolyl (BTH) and 2-(aminophenyl)benzothiazolyl (AP-BTH) C-terminal modified amino acids and peptides with high chiral purity.

## 1. Introduction

**Figure 1:**
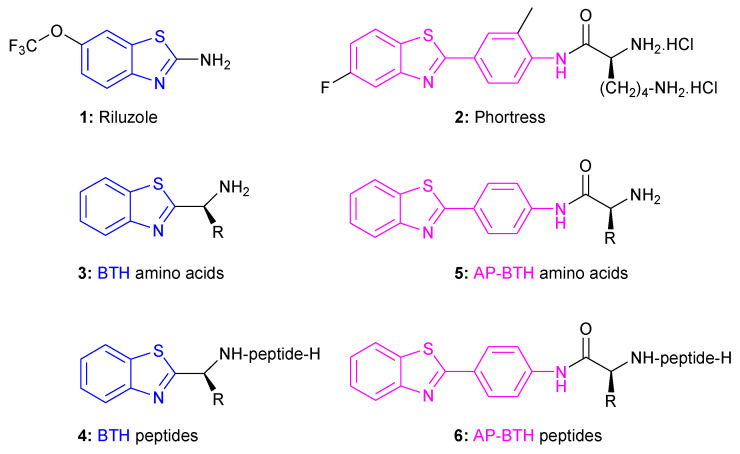
Representative 2-benzothiazolyl and 2-(aminophenyl)benzothiazolyl drugs (**1**, **2**) and the synthesized BTH (**3**, **4**) and AP-BTH (**5**, **6**) derivatives of these.

2-benzothiazoles (BT) represent an important class of compounds possessing a wide spectrum of biological activities, such as anti-inflammatory [1,2], antifungal [3], antiviral [4,5], analgesic [6], antioxidant [7,8], antipsychotic [9], anticonvulsant [10], antidiabetic [11,12], and anti-cancer activities [13,14]. Among thousands of BTs under investigation, Riluloze **1** and Phortress **2** are among the most representative therapeutic agents. Riluzole **1** is an anticonvulsant and neuroprotective FDA-approved drug to increase survival among patients with Amyotrophic Lateral Sclerosis (ALS), with potential as a novel anti-cancer agent [15,16]. Phortress **2** is a well-known antitumor agent, with potent and selective activity against human-derived carcinomas of breast, ovarian and renal origin (Figure 1) [17,18,19].

In addition to Riluzole and Phortress, other BT-type compounds have also demonstrated significant pharmacological activity. The best-known examples include Zopolrestat, a highly potent, orally active aldose reductase (AR) inhibitor currently in phase III clinical trials [20,21], Ethoxzolamide, an FDA-approved human carbonic anhydrase inhibitor, and Frentizole [22], an FDA-approved nontoxic antiviral and immunosuppressive drug, clinically used in rheumatoid arthritis and systemic lupus erythematosus, acting as an inhibitor of the interaction between amyloid beta peptide (Aβ) and amyloid-binding alcohol dehydrogenase (ABAD) [23]. From a structural point of view, Riluzole **1**, Zopolrestat, Ethoxzolamide and Frentizole contain the 2-benzothiazolyl (BTH) scaffold, while Phortress **2** contains the 2-(4-aminophenyl)benzothiazolyl (AP-BTH) scaffold [18,24,25].

Besides Phortress, the AP-BTH scaffold has shown remarkable biological properties and is considered a potent and selective pharmacophore and a scaffold of special interest, found in many antitumor, anti-Alzheimer and anti-microbial agents [19]. In addition to these properties, the high interaction ability of 2-(aminophenyl)benzothiazole scaffolds with amyloid fibrils, clearly indicated by Thioflavin-T, has led to the synthesis of 2-(4-aminophenyl)benzothiazole decorated nanovesicles that effectively inhibit A*β*_1–42_ fibril formation and exhibit in vitro brain targeting potential [26].

2-phenylbenzothiazoles have also shown interesting metal-involved noncovalent interactions [27,28,29,30] with broad applications in materials science, while in the diagnostic area, many metal-radiolabeled 2-phenylbenzothiazole derivatives are continuing to be explored as amyloid imaging agents [23,31,32,33]. Non-metal-based positron emission tomography (PET) imaging of Aβ plaques is also possible through the use of radiolabeled benzothiazoles such as the ^18^F-labeled derivative of 2-(4-aminophenyl)benzothiazole [^18^F] Flutemetamol, which gained approval in 2013 for clinical use [34,35]. Based on the high interest in BTHs and AP-BTHs, several benzothiazole derivatives and hybrids have also been designed [24,36,37]. In this direction, the combination of BTH and AP-BTH scaffolds with amino acids and peptides is of interest, as such compounds would combine the advantages of BTH/AP-BTH scaffolds with the advantages of amino acids/peptides. For this, suitable chemistries that would allow the easy and efficient synthesis of such compounds are of great interest. In particular, the use of Solid-Phase Synthesis (SPS) as a tool for the synthesis of tile compounds would greatly simplify their synthesis and the discovery of new bioactives [38].

2-benzothiazoles are synthesized via several methods, including the condensation of ortho-aminobenzenethiols with carboxylic acid derivatives, the radical cyclisation of thioacylbenzanilides, or the base-induced cyclization of the corresponding ortho-haloanilides [39,40,41]. These methods are performed in solution under conditions not appropriate for SPS and in several cases give complex product mixtures.

Regarding the synthesis of the 2-(4-aminophenyl)benzothiazole scaffold, this is mainly achieved through the cyclization of nitro-substituted thiobenzanilides to nitrophenyl-benzothiazoles using the Jacobson synthesis and subsequent reduction, while certain 2-(4-aminophenyl)benzothiazoles with no substituents in the benzothiazolyl moiety are prepared via the reaction of 2-aminobenzenethiol and 4-aminobenzoic acid or benzonitrile in polyphosphoric acid (PPA) at high temperatures (220 °C) [18]. The condensation of 2-(4-aminophenyl)benzothiazole with amino acids has been achieved by its reaction with 1-hydroxybenzotriazole activated amino acids [25]. Besides the limitations of the applied chemistries during 2-(4-aminophenyl)benzothiazole synthesis (high temperatures, complex product mixtures, substrate dependence) and the conjugation of 2-(4-aminophenyl)benzothiazole with 1-hydroxybenzotriazolyl amino acids (low reactivity, repeated coupling cycles, high reaction times) [18,25], such methods are not appropriate for the synthesis of AP-BTH peptides where SPS or Convergent Synthesis methods are required [42,43].

**Scheme 1:**
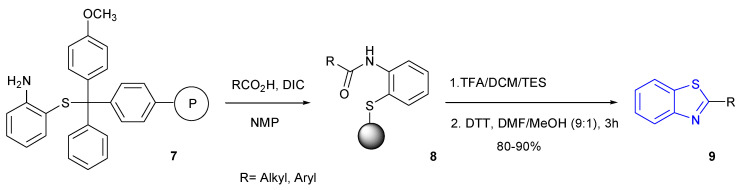
General method for the solid-phase synthesis of alkyl and aryl 2-benzothiazolyl compounds **9**.

In the present work we considered, for the first time, SPS as an easy and efficient approach for the synthesis of BTH amino acids and peptides (type **3** and **4**, respectively), as well as the synthesis of AP-BTH amino acids and AP-BTH peptides (type **5** and **6**, respectively), using resin-bound 2-aminobenzenethiol **7** (Scheme 1). This resin (**7**) has been effectively used by us for the SPS of 2-benzothiazolyl compounds [44]. In brief, 2-aminobenzenethiol was initially attached to trityl type resins, preferably 4-methoxytiryl (Mmt)-resin (resin **7**), and this resin was further used for the synthesis of several 2-alkyl and 2-arylbenzothiazoles **9**. These were obtained by the acylation of **7** with alkyl and aryl carboxylic acids, activated with *N,N*′-diisopropylcarbodiimide (DIC), to obtain the resin-bound 2-*N*-acyl-aminobenzenethiols **8**, which, upon treatment with mild trifluoroacetic acid (TFA) solutions in dichloromethane (DCM) and triethylsilane (TES), liberated the 2-*N*-acyl-aminobenzenethiols, which were cyclized into the corresponding 2-substituted benzothiazoles **9** (Scheme 1).

Taking advantage of this method, we considered the synthesis of BTH and AP-BTH amino acids and peptides by combining this methodology with the widely used 9-fluorenylmethoxycarbonyl (Fmoc)/tert-butyl (*^t^*Bu) strategy in Solid-Phase Peptide Synthesis (SPPS) [45]. The Fmoc/*^t^*Bu strategy requires mild conditions and an orthogonal protection system; amino acid side chains are protected by acid-labile groups, while the alpha amine group is protected by the base-labile Fmoc group. As the most suitable resin for our synthetic approach, we selected Mmt-resin **7** [44]. This resin would allow the synthesis of the side chain fully deprotected BTH and AP-BTH amino acids/peptides upon treatment with high concentrations of TFA solutions (65–90% TFA in the presence of scavengers, i.e., TES), while upon treatment with 1.1% TFA in the presence of scavengers, the side chain protected BTH and AP-BTH amino acids/peptides could be obtained. The key point of the proposed method is the coupling of the first amino acid to resin-bound 2-aminobenzenethiol **7**, due to the relatively low nucleophilicity of the aromatic amine group, thus a highly activated Fmoc-amino acid would be required. Obviously, the activating agents used for this reaction would influence not only the rate of reaction but also the degree of racemization of the first amino acid. For this, the degree of racemization of the first amino acid for BTH (and AP-BTH) amino acid derivatives (**3** and **5**) was measured and the results are discussed. Furthermore, in order to demonstrate the applicability of the proposed method, a series of BTH and AP-BTH amino acids/peptides (of types **4** and **6**) were synthesized.

## 2. Results and Discussion

### 2.1. Synthesis of BTH-Amino Acids ***3*** and Peptides ***4***

#### 2.1.1. General Method Analysis

For the synthesis of BTH-amino acids (AAs) **3** and ΒΤH-peptides **4**, resin-bound 2-aminobenzenethiol **7** was initially reacted with representative Fmoc-amino acids (Leu, Glu, Lys, Arg, Ser), including the most racemization-prone amino acids Cys and His. Common protecting groups for the orthogonal protection of these AAs were selected: *^t^*Bu, (tert-butyloxycarbonyl) Boc, (trityl) Trt, (2,2,4,6,7-pentamethyldihydrobenzofuran-5-sulfonyl) Pbf [46].

**Scheme 2:**
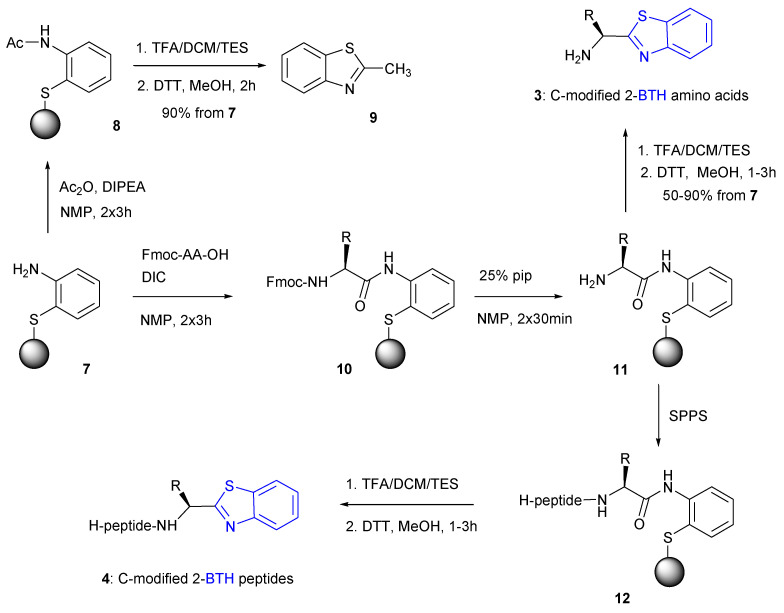
General method for the solid-phase synthesis of C-modified BTH-amino acids and peptides.

Thus, resin **7** was initially reacted for 3 h at rt with a five-molar excess of the Fmoc-amino acid in *N*-methyl-2-pyrrolidone (NMP) using DIC as the activating agent, leading to resin **10**, which upon treatment with 25% piperidine in NMP gave the Fmoc-deprotected resin **11** (Scheme 2).

Resin **11** was further treated with TFA in DCM/TES (95:5), which allowed cleavage from the resin and formation of the BTH-amino acids **3** after cyclization of the liberated 2-*N*-aminoacyl-aminobenzenethiols, within 1–3 h at rt, in methanol (MeOH) (or NMP/MeOH) in the presence of dithiothreitol (DTT) (0.1–0.2 mmol) (which acts as a reducing agent for any disulfides that might be formed during the cleavage process) [44]. The use of 1.1% TFA in DCM/TES (95:5) allowed the formation of fully protected BTH-amino acids, while treatment with higher concentrations of TFA (>65%) resulted in simultaneous cleavage from the resin and side-chain deprotection [46,47], allowing the formation of fully side-chain deprotected BTH-amino acids.

In case of an incomplete coupling between the first Fmoc-amino acid and resin **7** (which was easily identified by the presence of oxidized bis-2-aminothiophenol either by TLC or HPLC analysis of the cleavage mixture), a second coupling reaction was performed under the same conditions (prior to Fmoc deprotection). In case of a second incomplete coupling, the unreacted amines were blocked via acetic anhydride and *N,N*-diisopropylethylamine (DIPEA) through the formation of resin-bound 2-*N*-acetyl-aminobenzenethiol **8**. In this case, 2-methylbenzothiazole (BTH-CH_3_) **9** was also formed upon treatment with TFA/TES and cyclization (Scheme 2).

Regarding the coupling efficiency of the first amino acid, it was found that the use of a five-molar excess of the first Fmoc-amino acid resulted in relatively high coupling efficiencies, with almost complete coupling reactions (80–90%) in most cases, estimated according to the relative quantification of BTH-AAs **3** and (BTH-CH_3_) **9** in HPLC profile analysis of the fully deprotected mixture (after cleavage and cyclization; crude mixture). Fmoc-L-His(Trt)-OH was a special case, where moderate coupling yields were identified during the first coupling cycle (50–60%) with a small increment in the second coupling cycle (70–80%), and therefore, blocking the unreacted amine groups was necessary in this case.

Resin **11** was also applied in the synthesis of BTH-peptides **4** using SPPS methods and Fmoc/*^t^*Bu protected amino acids. DIC and (1-hydroxybenzotriazole) HOBt were used as the activation system to form resin-bound peptidyl 2-aminobenzenethiol resin **12**, which, upon treatment with the appropriate concentration of TFA/TES and subsequent cyclization (as previously described), resulted in either side-chain protected or deprotected BTH peptides **4** (depending on the acid strength of the cleavage mixture) (Scheme 2).

#### 2.1.2. Racemization during the First Fmoc-Amino Acid Coupling with Resin **7**

**Figure 2:**
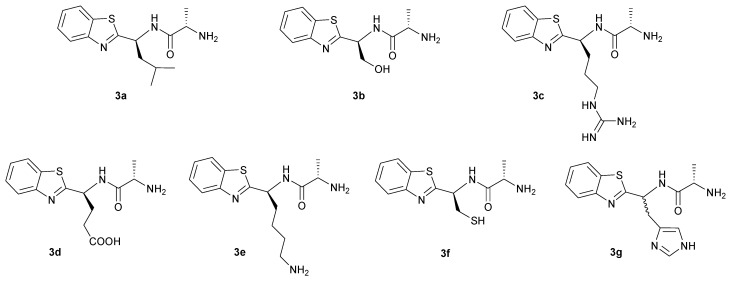
BTH dipeptides **3a**–**3g**, which were synthesized to measure the degree of racemization during the coupling reaction of the first Fmoc-amino acid with resin **7**.

An important parameter in the coupling of the first amino acid (AA1) is the expected racemization of the first amino acid due to the use of DIC (with no other coupling additives) as the condensing agent. In order to estimate the degree of racemization during the reaction of the first Fmoc-amino acid with resin **7**, we reacted resin **11** with Fmoc-L-Ala-OH, which resulted in the synthesis of the corresponding dipeptides H-L-Ala-L-AA1-BTH **3a**–**g** (after on-resin treatment with 25% piperidine) (Figure 2). These BTH-dipeptides, as well as their corresponding H-L-Ala-D-AA1-BTH diastereomers, which were also synthesized, enabled the measurement of the degree of racemization during the coupling reaction of the first amino acid.

For this, both H-L-Ala-L-AA1-BTH and H-L-Ala-D-AA1-BTH were subjected to HPLC analysis (separately injected and as a mixture) using appropriate conditions for their complete separation, paying special attention so that no undesired peaks interfered in the integration area, thus establishing optimal methods for the separation of the two diastereomers. This allowed us to estimate the chiral purity of the synthesized BTH-dipeptides (**3a**–**g**) via HPLC analysis of the synthesized H-L-Ala-L-AA1-BTH and subsequent integration of the two peaks: H-L-Ala-L-AA1-BTH (main diastereomer) and H-L-Ala-D-AA1-BTH (formed due to racemization). Due to the relatively low racemization values seen in most cases, in order to undisputedly evaluate the diastereomeric purity, spiking of separately synthesized H-L-Ala-D-AA1-BTH into the analyzed H-L-Ala-L-AA1-BTH was performed when needed. By this method, we were able to measure the degree of diastereomeric purity of the synthesized H-L-Ala-L-AA1-BTH, which determines the chiral purity of the corresponding H-L-AA1-BTH (no racemization is expected upon insertion of the second Fmoc-amino acid). The results of this investigation are summarized in Table 1.

As can be seen, the reaction of resin **7** with Fmoc-amino acids activated with DIC (conditions 1; Table 1) resulted in low racemization values, which were limited between 0.25% for Leu and Ser(*^t^*Bu) and 1.89% for the racemization prone Cys(Trt). In the cases of Glu(*^t^*Bu) and Lys(Boc) the racemization was 1.13% and 1.16%, respectively, while Arg(Pbf) gave a racemization of 0.41%. His(Trt) was a special case, as this amino acid resulted in considerable amounts of racemization (44.9%) when DIC was used as the condensing agent. In an effort to lower its racemization during the first amino acid coupling, we initially attempted to add DIC in a portion-wise manner to the reaction mixture; however, this practice did not significantly improve the degree of racemization (Table 1). In contrast, when 1-hydroxy-7-azabenzotriazole (HOAt)/DIC [48] was used as coupling agent, lower degrees of racemization were seen for all amino acids [Ser(*^t^*Bu) < 0.1%; Arg(Pbf) 0.32%; Glu(*^t^*Bu) 0.98%; Lys(Boc) 0.58%; Cys(Trt) 1.32%]; however, the coupling yields were relatively reduced (70–80%). In the case of His(Trt), the use of HOAt/DIC as the condensing agent gave considerably lower racemization values (7.64%); however, the coupling yield was limited to 40–50% even after two coupling cycles, and therefore blocking of the unreacted amine groups was necessary in this case. In addition, the use of O-[(cyano-(ethoxycarbonyl)methyliden)-amino]-yloxytripyrrolidinophosphonium hexafluorophosphate and tetrafluoroborate (pyOxim)/DIPEA [49] as an activating agent system for His(Trt) did not significantly lower the degree of racemization, which was measured at 24.9%, while the coupling yield was even lower (20–30% after two coupling cycles), and therefore this method of activation was not considered satisfactory, neither in terms of racemization nor in terms of coupling efficiency of the first Fmoc-amino acid with resin **7**. It should be noted that all reactions and measurements were performed twice, and the measured values were found to be repeatable and consistent with those reported. Representative analytical HPLC for quantification of the degree of racemization are presented in the Supplementary File (Figures S1–S14).

#### 2.1.3. Applicability in the Solid-Phase Synthesis of BTH-Amino Acid Libraries and BTH-Peptides

**Scheme 3:**
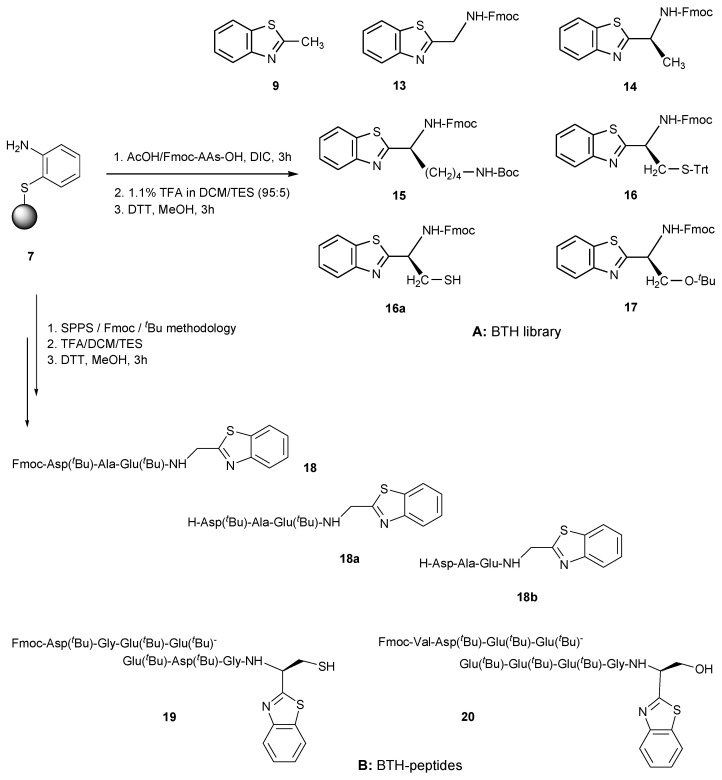
(**A**) BTH-amino acid library produced by the reaction of resin **7** with a mixture of AcOH, Fmoc-Gly-OH, Fmoc-L-Ala-OH, Fmoc-L-Lys(Boc)-OH, Fmoc-L-Cys(Trt)-OH, and Fmoc-L-Ser(*^t^*Bu)-OH in the presence of DIC as the condensing agent, and subsequent cleavage and cyclization of the corresponding BTH-amino acids (**9**, **13**, **14**, **15**, **16**, **16a**, **17**). (**B**) Examples of BTH-peptides that were synthesized: *^t^*Bu/Fmoc protected BTH-peptide **18**; *^t^*Bu-protected/Fmoc-deprotected BTH-peptide **18a**; *^t^*Bu-deprotected/Fmoc-deprotected BTH-peptide **18b**; Fmoc/*^t^*Bu-protected BTH-Cys peptide **19**; BTH-Ser-peptide **20**.

As the next step in this work, in order to further reveal the applicability of the proposed method in the solid-phase synthesis of BTH-amino acids and peptides, we synthesized a small BTH-amino acid library consisting of *^t^*Bu-type-protected/Fmoc-protected BTH-amino acids (**9**, **13**, **14**, **15**, **16**, **17**) and synthesized a series of BTH-peptides (**18**, **18a**, **18b**, **19**, **20**) (Scheme 3).

The BTH-amino acid library was prepared by reacting a mixture of an equimolar amount of AcOH, Fmoc-Gly-OH, Fmoc-L-Ala-OH, Fmoc-L-Lys(Boc), Fmoc-L-Cys(Trt)-OH, and Fmoc-L-Ser(*^t^*Bu)-OH with resin **7** using DIC as the condensing agent. Cleavage from the resin with 1.1% TFA in DCM/TES (95:5) and cyclization in DTT resulted in the side-chain protected BTH-amino acid library in Scheme 3 (Figure S15). It should be noted that 4–5% of S-Trt-deprotected BTH-Cys-H **16a** was identified in the product mixture, a known result of treatment of Cys(Trt) with 1.1–1.5% TFA/TES [47,50,51].

In addition, we synthesized BTH-peptides **18**, **18a**, **18b**, **19** and **20** (Scheme 3). BTH-peptide **18** was synthesized using Fmoc-Gly-OH as the first amino acid and Fmoc-L-Glu(*^t^*Bu)-OH, Fmoc-L-Ala-OH and Fmoc-L-Asp(*^t^*Bu)-OH as the next amino acids. Treatment of the peptidyl resin with 1.1% TFA in DCM/TES (95:5) and cyclization of the liberated 2-*N*-peptidyl-aminobenzenethiol (as previously described) resulted in the formation of the side-chain-protected/Fmoc-protected BTH-peptide **18** (Figure S16A). When Fmoc was removed (prior to cleavage from the resin), the corresponding *^t^*Bu-protected/Fmoc-deprotected BTH-peptide **18a** was obtained (Figure S16B), while further treatment of **18a** with TFA/DCM/TES 90/5/5 resulted in the formation of the fully side-chain-deprotected and *N*-Fmoc-deprotected **18b** (Figure S16C). 

Similarly, the BTH-peptides **19** and **20** were synthesized via use of acid labile Fmoc-L-Cys(Mmt)-OH (**19**) and Fmoc-L-Ser(Trt)OH (**20**) as the first amino acids and subsequent SPPS. Treatment of the synthesized peptidyl resins with 1.1% TFA/TES in DCM/TES (95:5) and subsequent cyclization allowed simultaneous cleavage from the resin and removal of the *S*-Mmt/O-Trt protecting groups, which resulted in the preparation of the corresponding BTH-Cys-peptide **19** (Figure S17A) and BTH-Ser-peptide **20** (Figure S17B).

It should be highlighted that **18a**, **19** and **20** are side-chain protected BTH-peptides, and they all possess one free active group (**18a** an amine group, **19** a thiol group, **20** a hydroxyl group), which allows their further use in solid-phase or liquid phase methods for synthesis of BTH-modified peptides through fragment condensation/convergent synthesis methodologies [42,45]. A scaled-up synthetic protocol for **18**, **18a** and **18b** (described in Section 3.3.6) resulted in high yields of the corresponding BTH-peptides, revealing the effectiveness and scalability of the proposed methods.

### 2.2. Synthesis of AP-BTH-Amino Acids ***5*** and Peptides ***6***

#### 2.2.1. General Method—Coupling of Aminobenzoic Acids to Resin **7**

Prompted by the positive results for BTH-amino acids/peptides and the pharmacological significance of the 2-(aminophenyl)benzothiazolyl (AP-BTH) scaffold, we considered of special interest the extension of this method for the synthesis of AP-BTH amino acids **5** and peptides **6**. The key points for this synthesis would be the successful introduction of the aminobenzoic acid scaffold to resin **7**, the subsequent successful introduction of the first amino acid (with low/minimum degree of racemization), and cyclization (after cleavage from the resin) into the AP-BTH scaffold.

**Scheme 4:**
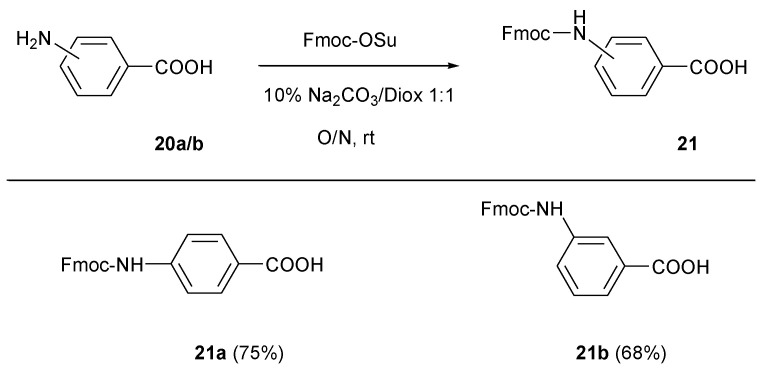
Synthesis of 4-(Fmoc-amino)benzoic acid **21a** and 3-(Fmoc-amino)benzoic acid **21b**.

For this, we initially synthesized *N*-Fmoc-3-aminobenzoic acid **21a** and *N*-Fmoc-4-aminobenzoic acid **21b** (Scheme 4) via the reaction of commercially available 3- and 4-aminobenzoic acids **20a/b** with Fmoc-OSu in 10% Na_2_CO_3_/Dioxane (^1^H, ^13^C-NMR of the synthesized products **21a/b** are presented in Figures S18–S21, while their analytical HPLC spectra are presented in Figures S22A and S23A).

In order to condense **21a/b** with resin-bound 2-aminobenzenethiol **7**, we initially tested the use of a five-fold molar excess of **21a/b** over **7** using DIC as the condensing agent in NMP. Unfortunately, this reaction was unsuccessful, with no condensation product identified by HPLC analysis, possibly due to the reduced reactivity of the aromatic O-acylisourea in **21a/b** and reduced nucleophilicity of the aromatic amine group in resin **7**, as well as the formation of the corresponding *N*-acylurea by-product of **21a/b** (which was finally identified in the reaction mixture through HPLC and ESI-MS analysis) due to the so-called O,N-acyl migration [52]. The use of HOAt as an additive did not improve the reaction yield, and no product was identified in this case either.

**Scheme 5:**
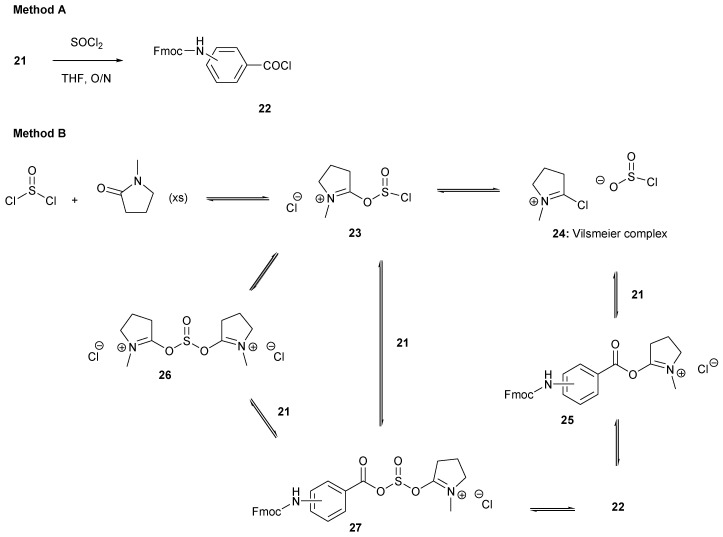
(**A**) Chlorination of Fmoc-4-aminobenzoic acid (**21a**) and Fmoc-3-aminobenzoic acid (**21b**) with SOCl_2_ (5 eq; overnight reaction at rt) in THF (Method A); (**B**) Proposed mechanism for the catalytic activity of NMP during activation of Fmoc-4-aminbenzoic acid (**21a**) and Fmoc-3-aminobenzoic acid (**21b**) in SOCl_2_ (1.02 eq) and 5% NMP in THF or DCM (5–30 min reaction at rt) (Method B).

**Scheme 6:**
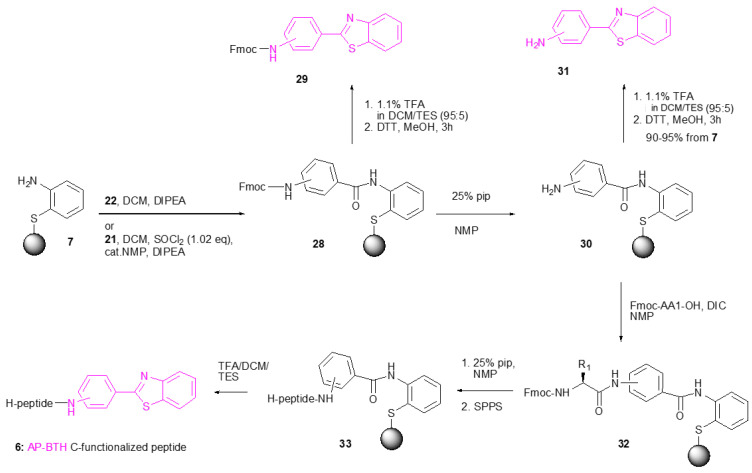
General method for the solid-phase synthesis of C-modified AP-BTH amino acids and peptides.

As an alternative route, we considered the formation of the corresponding acyl chlorides of **21a/b**. Therefore, we initially tested the reaction of **21a/b** with thionyl chloride (SOCl_2_) in toluene or DCM. Unfortunately, the reaction did not proceed with either of these two solvents, possibly due to the very low solubility of **21a/b** (in both solvents **21a/b** were not dissolved/did not react even after prolonged reaction times). However, when the reaction solvent was changed to THF, **21a/b** were completely soluble and we were able to prepare acyl chlorides **22a/b** after overnight reaction at rt by the use of a five-fold molar excess of SOCl_2_ relative to **21a/b** (Scheme 5A). It should be noted that the chlorination reaction proceeded at a slow reaction rate, and a second five-molar excess of SOCl_2_ was needed to complete chlorination_._ The reaction rate was easily followed using TLC and/or HPLC analysis to detect the formation of the corresponding methyl ester after quenching the reaction mixture with MeOH (analytical HPLC of **21a/b** and the corresponding methyl esters are presented in Figures S22A,B and S23A,B). In order to further react acyl chlorides **22a/b** with resin **7**, the reaction mixture was condensed until an oily product was formed. This was re-dissolved in toluene and further condensed using three vacuum distillation cycles to completely remove excess SOCl_2_, until a white solid (**22a/b**) was finally formed. This was initially dissolved in DCM and then added to resin **7**, which was pre-suspended in DCM and DIPEA, to afford **28** (which was identified by the formation of **29** (Scheme 6)).

In an effort to simplify the chlorination process, we considered another method for the activation of **21a/b** based on the reaction of **21a/b** with SOCl_2_ in THF or DCM using 5% NMP (*v*/*v*) as catalyst. In fact, the addition of 5% NMP to the reaction mixture of **21a** and SOCl_2_ (1.02 eq) in DCM resulted in fast activation of **21a** (less than 5 min at rt) (evidenced by the fast dissolution of **21a**, initially insoluble in DCM), also proved by quenching a small sample of the activated **21a** with MeOH, which resulted in the formation of the corresponding methyl ester. The effective activation of **21a** in the presence of NMP was easily followed with HPLC analysis, where the HPLC profile of the reaction of **21a** with SOCl_2_ in DCM before the addition of NMP (Figure S22A) showed the existence of only **21a**, while 5 min after the addition of 5% NMP (*v*/*v*), quenching of the reaction mixture with MeOH resulted in the formation of the corresponding methyl ester (Figure S22C) (a clear indication of the successful activation of **21a**).

It should be noted that the activation of **21b** using an equimolar amount of SOCl_2_ (1.02 mmol) and 5% NMP required longer reaction times compared with **21a**. In fact, the activation of **21b** was performed in THF/DCM (2:1), wherein the effective activation of **21b**, when NMP was added to the reaction mixture, was completed in 20–30 min at rt. The effective activation of **21b** in the presence of NMP was also confirmed by HPLC analysis, where the initial **21b** (Figure S23A) was completely converted into the corresponding methyl ester by quenching a sample of the reaction mixture with MeOH (Figure S23C).

As an explanation of the fast activation of **21a/b** in the presence of NMP, in contrast to the very slow chlorination reaction in absence of NMP, we considered the catalytic activity of NMP, as evidenced by the fast activation of **21a/b** when NMP was added to the reaction mixture, and therefore we propose the mechanism in Scheme 5B. Although the proposed mechanism has not yet been clarified, NMP has been proposed as a catalyst for the reaction of carboxylic acids with SOCl_2_, possibly through the formation of **23**, leading to the Vilsmeier complex **24** and/or **26** [53,54,55,56,57,58,59]. Thus, the reaction of **23** and/or **26** with **21a/b** would form **27**, while the reaction of **24** with **21a/b** would give **25**. In addition, the equilibrium of **25** and/or **27** with **22** is another possible route for the fast activation of **21a/b**, based on the nucleophilic attack of the chloride anion on the activated carbonyl groups of **25** and/or **27**, resulting in the corresponding acyl chloride **22** through a different route of activation (through **25** and/or **27**). Although this is a proposed mechanism, the presence of **25** and/or **27** and/or **22**, through the catalytic activity of NMP, is proved by the fast activation of **21a/b** only when NMP is added to the chlorination mixture. In any case, this procedure allowed the fast activation of the carboxylic acid group of **21a/b** (as confirmed by the fast reaction of the activated **21a/b** with excess MeOH yielding the corresponding methyl esters).

As the next step in our synthetic approach, the activated **21a/b** were reacted with resin **7** in the presence of DIPEA at rt to form **28** (Scheme 6) and the corresponding Fmoc-deprotected **30**. It should be noted that when activation was carried out with SOCl_2_ (1.02 eq) and 5% NMP (Method B), the synthesis of **28** (and **30**) was greatly simplified, compared with the activation of **21a/b** with excess SOCl_2_ (in the absence of NMP), as no extra work-up was needed and therefore the formation of AP-BTH **29** proceeded using this protocol with exceptional ease and purity (Figures S22D and S23D). To further ensure the efficacy of the proposed protocol, 2-(4-aminophenyl)benzothiazole (4-AP-BTH; **31a**) and 2-(3-aminophenyl)benzothiazole (3-AP-BTH; **31b**) (Scheme 6) were also obtained through the acidic treatment of resin **30** and subsequent cyclization (the procedure is analytically described in Section 3.3.6). Both products were obtained in high yield (>90%) and purity (Figures S24–S27).

#### 2.2.2. Racemization during the First Fmoc-Amino Acid Coupling to Resin **30**

Based on the positive results from the reaction of Fmoc-amino acids with resin **7** (Section 2.1.2), as well as the low degree of racemization measured during the first coupling even when only DIC was used as the condensing agent (Section 2.1.2; Table 1), we considered the same approach as a method to couple the first amino acid to resin-bound 2-*N*-aminobenzoyl-aminobenzenethiol **30** (Scheme 6). Thus, resin **30** was reacted with Fmoc-amino acids in NMP using DIC as the condensing agent, which resulted in the formation of **32**. Removal of the *N*-Fmoc group with 25% piperidine in NMP gave, after treatment with TFA/TES, the corresponding AP-BTH amino acids **5**. In addition, SPPS methods afforded AP-BTH peptides **6** after acidic treatment with TFA/TES of the synthesized resin **33** (Scheme 6).

**Figure 3:**
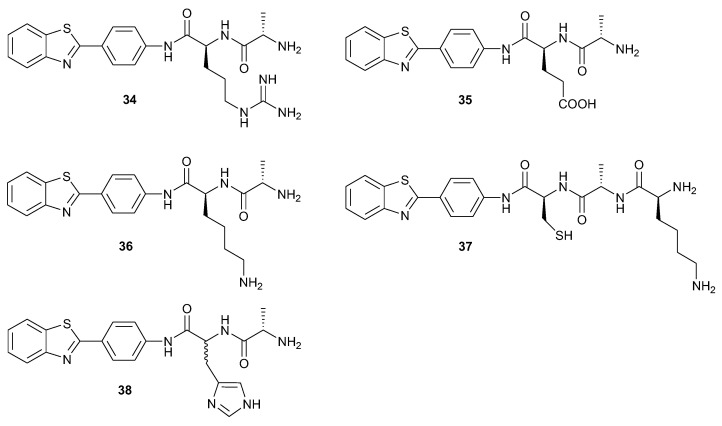
AP-BTH peptides (**34**–**38**) synthesized to measure the degree of racemization during the coupling reaction of the first Fmoc-amino acid with resin **30**.

In order to measure the degree of racemization during the coupling of the first Fmoc-amino acid with resin **30**, we considered Fmoc-L-Arg(Pbf)-OH, Fmoc-L-Lys(Boc)-OH, Fmoc-L-Glu(*^t^*Bu)-OH, Fmoc-L-Cys(Trt)-OH and Fmoc-L-His(Trt)-OH as representative amino acids (also used for the BTH-AAs racemization study, Section 2.1.2). The degree of racemization, which reflects the chiral purity of the synthesized AP-BTH amino acids, was evaluated through formation of the diastereomeric dipeptides H-L-Ala-L-AA1-AP-BTH and H-L-Ala-D-AA1-AP-BTH according to HPLC analysis (as clearly described in Section 2.1.2). In the case of Cys, separation of the two diastereomers was achieved through the synthesis of the tripeptide H-L-Lys-L-Ala-L-Cys-AP-BTH (and the corresponding H-L-Lys-L-Ala-D-Cys-AP-BTH). The structures that were synthesized for this study are shown in Figure 3, while analytical HPLC profiles measuring the degree of racemization are presented in the Supplementary File (Figures S28–S37). The results are presented in Table 2.

As can be seen in Table 2, the reaction of the first Fmoc-amino acid with resin **30** using DIC as the activating agent proceeded with considerably low racemization, where Arg(Pbf), Glu(*^t^*Bu) and Lys(Boc) showed exceptionally low racemization (<0.25%), while the racemization of Cys(Trt) was measured at 1.08%. H-His(Trt)-AP-BTH behaved similarly with H-His(Trt)-BTH (Section 2.1.2; Table 1) and therefore the initial degree of racemization of 44.5% (when DIC was used as the condensing agent) was finally limited to 7.65% (when HOAt/DIC was used as the condensing agent). It should be noted that all reactions and measurements were performed twice, and the measured values were found to be repeatable and consistent with those reported.

#### 2.2.3. Applicability in the Solid-Phase Synthesis of AP-BTH Peptides

**Figure 4:**
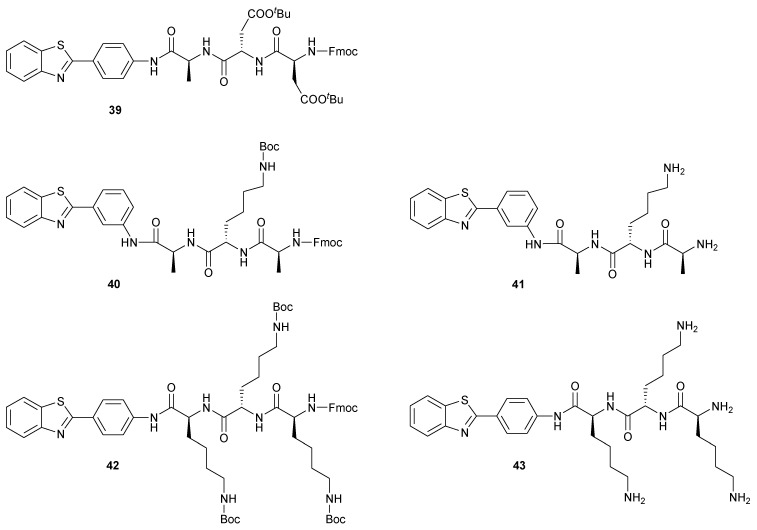
AP-BTH tripeptides of type **6** that were synthesized.

As a proof of concept, besides the racemization study, we also synthesized a small series of AP-BTH peptides (**39**–**43**; Figure 4). AP-BTH peptides **40** and **41** contain the 3-AP-BTH scaffold, while **39**, **42** and **43** contain the 4-AP-BTH scaffold. For the synthesis of **39**, **40**, and **42**, which are side-chain-protected AP-BTH peptides, the corresponding resins were treated with 1.1% TFA/TES, while for the synthesis of side-chain deprotected AP-BTH peptides **41** and **43**, the resins were treated with TFA/DCM/TES (90/5/5).

All AP-BTH peptides (**39**–**43**) were synthesized in high purity (the analytical HPLC spectra are presented in Figure S38), while their expected structures were successfully identified using ESI-MS. In addition, scaled-up synthesis of **42** and **43** (described in Section 3.3.6) gave high yields for both products, revealing the effectiveness and scalability of the method.

## 3. Materials and Methods

### 3.1. Materials

4-aminobenzoic acid 99% and 3-aminobenzoic acid 99+% were purchased from Thermo Scientific Chemicals (Acros) (Geel, Belgium). 1-hydroxy-7-azabenzotriazole (HOAt) and O-[(cyano-(ethoxycarbonyl)methyliden)-amino]-yloxytripyrrolidinophosphonium hexafluorophosphate (pyOxim) were purchased from Aapptec LLC (Louisville, KY, USA). 4-methoxytrityl chloride resin 100–200 mesh (loading capacity 1.0–2.0 mmol/g) and Fmoc-protected amino acids were provided by CBL Patras S.A. (Industrial area of Patras, Building block 1, GR-25018, Patras, Greece). All other chemicals were purchased from Sigma-Aldrich O.M. Ltd. (Athens, Greece). All chemicals were used without further purification.

### 3.2. Analytical Methods

Thin layer chromatography (TLC) was performed on precoated silica gel 60 F_254_ plates (Merck, Darmstadt, Germany) and spot detection was carried out using UV light and/or by charring with a ninhydrin solution. High Performance Liquid Chromatography (HPLC) analysis was performed on a Waters 2695 multisolvent delivery system (Milford, MA, USA), combined with a Waters 991 photodiode array detector. The following columns were used: (A) Lichrosphere RP-8, 5 μm, 125–4 mm; (B) Zorbax SB-C18, 3.5 μm, 30–2.1 mm; (C) Column: Lichrosphere RP-18, 5 μm, 125–4 mm; (D) YMC-Triart C18, 12 nm, S-5 µm, 250–4.6 mm. ESI-MS spectra were recorded on a Waters Micromass ZQ 4000 mass detector (positive mode), controlled using MassLynx 4.1 software (Milford, MA, USA), by direct infusion using a syringe pump at a flow rate of 5 mL/min. Cone voltage was set at 30 V and scan time at 1 s, with the interscan delay at 0.1 s. NMR spectra were recorded on a Brucker DPX 600 MHz instrument (Peoria, IL, USA). The sample spectra were recorded at 25 °C. Chemical shifts (δ) were referenced to the corresponding solvent peaks and are reported in parts per million (ppm). Coupling constants (*J*) are given in Hertz. Multiplicities are abbreviated as: s (singlet), d (doublet), t (triplet), q (quartet), m (multiplet), or combinations thereof.

### 3.3. Synthetic Procedures

#### 3.3.1. Synthesis of Fmoc-4-aminobenzoic Acid **21a** and Fmoc-3-aminobenzoic Acid **21b**

4-Aminobenzoic acid **20a** (or 3-aminobenzoic acid **20b**) (0.0146 mmol; 2 g) was placed in a round-bottom flask and the solid was dissolved in a mixture of dioxane and aq. 10% Na_2_CO_3_ (1:1) (40 mL). The mixture was kept under stirring at rt. To the resulting solution, *N*-(9-fluorenylmethoxycarbonyloxy)succinimide (Fmoc-OSu) (0.0160 mmol; 5.41 g) dissolved in 20 mL dioxane was slowly added and the pH of the reaction was periodically adjusted to around 8.0–9.0 using aq. 10% Na_2_CO_3_. The reaction mixture was further stirred overnight at rt, where a gradual increase in the formation of a white precipitate (**21a**/**b**) was observed. The reaction progress and completion was monitored via TLC analysis (until all starting materials **20a**/**b** were reacted). Then, ethyl acetate (EtOAc) was added (40 mL) and to this mixture conc. HCl was slowly added until reaching pH 2.0. The two phases were separated, and the aqueous phase was washed twice with EtOAc. The combined organic phases were washed with water (3 × 50 mL) and the organic phase was concentrated into a rotary evaporator, where a white solid was formed. This was obtained by washing with diethyl ether (DEE) (3 × 50 mL) and drying in vacuo. Yield: **21a** (3.93 g; 75%); **21b** (3.56 g; 68%). Purity: **21a** (>98%); **21b** (>98%) based on HPLC analysis (265 nm). **21a**: ^1^H-NMR *δ* (600 MHz, DMSO-*d*_6_) 4.33 (t, *J* = 6.4 Hz, 1H), 4.53 (d, *J* = 6.1 Hz, 2H), 7.35 (t, *J* = 7,4 Hz, 2H), 7.43 (t, *J* = 7.4 Hz, 2H), 7.55 (s, 2H), 7.75 (d, *J* = 7.4 Hz, 2H), 7.85 (d, *J* = 8.2 Hz, 2H), 7.91 (d, *J* = 7.5 Hz, 2H), 10.05 (s, 1H), 12.64 (s, 1H); ^13^C-NMR δ (150 MHz, DMSO-*d*_6_) 47.02, 66.25, 117.90, 120.65, 124.92, 125.54, 127.60, 128.17, 130.86, 141.27, 143.67, 144.15, 153.17, 167.41; ESI-MS *m*/*z* [M + H^+^] found to be 360.38; NMR analysis of **21a** was consistent with bibliographic data on **21a** synthesized via a different procedure [60,61]; **21b**: ^1^H-NMR *δ* (600 MHz, DMSO-*d*_6_) 4.32 (t, *J* = 6.7 Hz, 1H), 4.5 (d, *J* = 6.6 Hz, 2H), 7.45–7.33 (m, 5H), 7.57 (d, *J* = 7.6 Hz, 1H), 7.76 (d, *J* = 7.76, 2H), 7.91 (d, 7.5 Hz, 2H), 8.13 (s, 1H), 9.91 (s, 1H), 12.94 (s, 1H); ^13^C-NMR δ (150 MHz, DMSO-*d*_6_) 47.07, 66.16, 119.46, 120.67, 122.85, 123.80, 125.61, 127.61, 128.18, 129.46, 131.86, 139.80, 141.28, 144.21, 153.89, 167.64; ESI-MS *m*/*z* [M + H^+^] found to be 360.24.

#### 3.3.2. Synthesis of 2-Aminobenzethiol-4-methoxytrityl Resin (**7**)

An amount of 3.0 g of 4-methoxytrityl chloride resin (1.2–2.0 mmol/g; 200–400 mesh, 1% DVB) was suspended in DCM (5 mL/gr; 15 mL) and 2-aminobenzenethiol (1.5 mmol/g; 4.5 mmol; 469.46 μL) was added to the resin. The mixture was gently agitated for 2 h at rt and then filtered and sequentially washed with DCM (×2), DCM/ MeOH/ DIPEA 85:10:5 (3 × 15 min), NMP (×5), isopropyl alcohol (iPrOH) (×3), and DEE (×2) and dried in vacuo. Loading of the resin was estimated through two methods: (A) weight gain, by using the following formula: S(wt) = [Wt(g) × 1000]/[Wt(add) × Wt(t)], where S(wt): weight gain substitution (mmol/g); Wt(g): weight gained by resin (g); Wt(add): molecular weight added to the resin = MW of amino acid minus MW of leaving group (g/mol); Wt(t): total weight gain of the resin after loading (g). (B) HPLC analysis of resin **7** coupled with Fmoc-Gly-OH, subsequent cleavage of the Fmoc group with 25% piperidine and cyclization into H-Gly-BTH (by treating the resin with DCM/TFA/TES 90/5/5 to ensure fast and irreversible cleavage), concentration of the cleavage mixture and dissolution of the oil in NMP and subsequent cyclization with DTT. The absorbance at 254 nm was compared with the absorbance of a calibration curve of H-Gly-BTH, synthesized and isolated independently. Both methods (A and B) gave a loading of 0.4–0.5 mmol/g.

#### 3.3.3. Solid-Phase Synthesis—General Protocols

Solid-phase peptide synthesis was carried out manually either in plastic reactors for peptide synthesis (polypropylene syringes equipped with porous polyethylene frits at the bottom) with a pore size of 25 μm (obtained from Carl Roth Gmbh + Co. KG, Karlsruhe, Germany), attached to a Visiprep Solid Phase Extraction Vacuum Manifold, or in eppendorfs (where the reaction mixture was transferred to microfilters for washing).

##### 3.3.3.1. Coupling of the First Fmoc-Amino Acid with Resins **7** and **30**

(a) Activation with DIC

The appropriate Fmoc-amino acid (0.5 mmol) was dissolved in a minimum amount of NMP (5 mL/g resin; 1.0 mL) and cooled at 4 °C for 15 min. To this solution DIC (0.55 mmol; 86.12 μL) was added and the mixture was vortexed for 1 min and added to the pre-swollen resin (0.5 mmol/g; 0.1 mmol available amine groups; 0.2 g). The resin was gently agitated for 3 h at rt and then washed with NMP (×5). In cases of incomplete coupling (determined by washing a small quantity of the resin and performing TLC and/or HPLC analysis, where bis-2-aminobenzenethiol was identified) recoupling was performed via the same procedure with fresh reagents. Any unreacted amine groups were capped using DIPEA (0.33 mmol; 57.48 μL) and acetic anhydride (Ac_2_O) (0.3 mmol; 28.36 μL) in NMP (5 mL/g; 1.0 mL) for 3 h at rt. Finally, the resin was washed with NMP (×5), iPrOH (×3), DEE (×2) and dried in vacuo.

(b) Activation with HOAt/DIC

The appropriate Fmoc-amino acid (0.5 mmol) and HOAt (0.55 mmol; 0.075 g) were dissolved in a minimum amount of NMP (5 mL/g resin; 1.0 mL) and cooled at 4 °C for 15 min. Then, DIC (0.5 mmol; 78.29 μL) was added and the mixture was stirred for 20 min at 4 °C and then added to the pre-swollen resin (0.5 mmol/g; 0.1 mmol available amine groups; 0.2 g), and the resin was gently agitated for 4 h at rt and then washed with NMP (×5). In cases of incomplete coupling (as determined by TLC and/or HPLC analysis, where bis-2-aminobenzenethiol was identified) recoupling was performed via the same procedure. Any unreacted amine groups were capped using DIPEA (0.33 mmol; 57.48 μL) and Ac_2_O (0.3 mmol; 28.36 μL) in NMP (5 mL/g; 1.0 mL) for 3 h at rt. Finally, the resin was washed with NMP (×5), iPrOH (×3), and DEE (×2) and dried in vacuo.

(c) Activation with pyOxim/DIPEA

The appropriate Fmoc-amino acid (0.5 mmol) and pyOxim (0.5 mmol; 0.26 g) were dissolved in NMP (5 mL/g resin; 0.5 mL) and the mixture was cooled at 4 °C for 15 min. Then, DIPEA (1 mmol; 174.19 μL) was added and the mixture was activated for 1–2 min at 4 °C and then added to the pre-swollen resin (0.5 mmol/gr; 0.1 mmol available amine groups; 0.2 g). The resin was gently agitated for 4 h at rt and then washed with NMP (×5). In cases of incomplete coupling (as determined by washing a small quantity of the resin and TLC and/or HPLC analysis, where bis-2-aminobenzenethiol was identified) recoupling was performed via the same procedure. Any unreacted amine groups were capped using DIPEA (0.33 mmol; 57.48 μL) and Ac_2_O (0.3 mmol; 28.36 μL) in NMP (5 mL/g; 1.0 mL) for 3 h at rt. Finally, the resin was washed with NMP (×5), iPrOH (×3), and DEE (×2) and dried in vacuo.

##### 3.3.3.2. Coupling of **21a**/**b** with 2-Aminobenzethiol-4-methoxytrityl Resin (**7**)

(a) Synthesis of 4-(Fmoc-amino)benzoyl chloride (**21a**) and 3-(Fmoc-amino)benzoyl chloride (**21b**) in SOCl_2_ AND coupling with resin **7**.

**21a/b** (0.301 mmol; 0.108 g) were dissolved in tetrahydrofuran (THF) (1.0 mL), and then thionyl chloride (SOCl_2_) (5 eq; 1.504 mmol; 109.22 μL) was added and the reaction mixture was stirred at rt. After 3 h, a second amount of SOCl_2_ (109.22 μL) was added and the reaction mixture was stirred overnight until completion of chlorination. The reaction progress was followed using TLC and HPLC analysis by taking a small sample of the reaction mixture, which was then quenched with MeOH to form the corresponding **21a**/**b** methyl ester. Completion of the reaction was monitored according to the full conversion of **21a**/**b** to the corresponding methyl ester. Then, the chlorination reaction mixture was concentrated until an oily product was formed. This was further dissolved in toluene and removed for three vacuum distillation cycles to completely remove excess SOCl_2_ until a white solid (**22a**/**b**) was formed. This was initially dissolved in DCM, and then added to resin **7** (0.5 mmol/g; 0.2 g), which was suspended in a minimum volume of DCM and DIPEA (0.451 mmol; 78,58 μL). The resin was agitated for 3 h at rt, and then it was filtered and washed with NMP (×5), iPrOH (×3), and DEE (×2) to afford **28** (which was identified by the formation of **29**).

(b) Activation of **21a/b** using SOCl_2_ in THF/5% NMP AND coupling with resin **7**

**21a** (0.301 mmol; 0.108 g) was dissolved in DCM (or THF) (1.0 mL) and SOCl_2_ (0.307 mmol; 22.28 μL) was added. To this solution, NMP (5% *v*/*v*; 50 μL) was added and the reaction mixture was stirred for 5 min at rt. To confirm the activation of **21a**, TLC and HPLC analysis were used by taking a small sample of the reaction mixture, which was quenched with MeOH to form the corresponding **21a** methyl ester.

For the activation of **21b** (0.301 mmol; 0.108 g), this was dissolved in a mixture of THF/DCM (2:1) (1.5 mL), and then SOCl_2_ (0.307 mmol; 22.28 μL) was added. To this, NMP (5% *v*/*v*; 50 μL) was added and the reaction mixture was stirred for 30 min at rt. The successful activation of **21b** was also confirmed through TLC and HPLC analysis by taking a small sample of the reaction mixture, which was quenched with MeOH to form the corresponding **21b** methyl ester.

The activated **21a**/**b** were added to resin **7** (0.5 mmol/g; 0.2 g), which was pre-swollen with the minimum amount of DCM, and also contained the appropriate amount of DIPEA (0.451 mmol; 78.58 μL). The resin was agitated for 3 h at rt, and then it was filtered and washed with NMP (×5), iPrOH (×3), and DEE (×2) to afford **28** (which was identified by the formation of **29**).

##### 3.3.3.3. Coupling of Fmoc-Amino Acids with HOBt/DIC—Peptide Assembly

The appropriate Fmoc-amino acid (0.3 mmol) and HOBt (0.45 mmol) were dissolved in NMP (0.5 mL) and the mixture was cooled at 4 °C for 15 min. Then, DIC (0.36 mmol) was added and the mixture was agitated for 15 min at 4 °C, and further added to the resin-bound amino acid or peptide (0.1 mmol). The resin was agitated for 3 h at rt. After this time, the completion of the reaction was checked via the Kaiser test. Briefly, a sample of the resin was taken and washed with NMP, iPrOH, and DEE several times and then transferred to a small glass tube where 2 drops of each of the Kaiser solutions was added and the glass was heated to 120 °C for 4–5 min. A positive Kaiser (blue resin beads) was an indication of incomplete coupling, while a negative Kaiser test (colorless/yellowish beads) was an indication of complete coupling. In cases of incomplete coupling, recoupling was performed with a fresh solution of activated Fmoc-amino acid. Finally, the resin was filtered and washed with NMP (×5), iPrOH (×3), and DEE (×2) and dried in vacuo.

##### 3.3.3.4. Fmoc Removal during Solid-Phase Peptide Assembly

Resin-bound Fmoc-protected amino acids or peptides were initially washed with NMP (6 mL/gr) (5 times) and then treated with 25% piperidine in NMP (6 mL/gr) for 30 min at rt (twice). To check the completion of Fmoc removal, two tests were applied. Initially, a positive Kaiser test indicated removal of the Fmoc group, while to be sure that all Fmoc has been removed, a second test was performed where a resin probe (approx. 2 mg) was treated with 25% piperidine in NMP (20 μL) and the resin was heated for 5 min at 100 °C. From the resulting solution 10 μL were spotted onto a TLC plate and run a few centimeters (and checked under a UV lamp for any UV-absorbing material). In cases of Fmoc absorbance, the deprotection was repeated for another 30 min. Finally, the resin was filtered and washed with NMP (×5), iPrOH (×3), and DEE (×2) and dried in vacuo.

#### 3.3.4. General Procedures for the Acidic Cleavage and Subsequent Cyclization to BTH-and AP-BTH-Amino Acids and Peptides

##### 3.3.4.1. Cleavage and Cyclization into Side-Chain *^t^*Bu-Protected/*N*-Terminus Fmoc-Protected BTH and AP-BTH Amino Acids and Peptides (**9**, **13**, **14**, **15**, **16**, **16a**, **17**, **18**, **19**, **20**, **39**, **40**, **42**)

Resin-bound *^t^*Bu-protected/Fmoc-protected derivatives were pre-treated with 0.1% TFA in DCM (×2) and the filtrates were discarded. Then, the resin was treated with 1.1% TFA in DCM/TES (95:5) for 15 min at rt and the cleavage mixture was filtered and further washed twice with a fresh cleavage mixture. The combined filtrates were concentrated on a rotary evaporator until an oily product was formed, and then two methods were developed:

(a) The oily products (fully protected derivatives) were dissolved in MeOH and DTT (0.1–0.2 eq) was added, and the mixture was stirred for 1–3 h at rt to allow cyclization into BTH and AP-BTH amino acid/peptide derivatives (completeness of cyclization was monitored using HPLC analysis). Then, MeOH was removed and the oily product that was formed was washed with either DEE or a mixture of DEE/hexane (Hex) (or Hex), and the product was dried in vacuo.
(b) The oily products (fully protected derivatives) were dissolved in MeOH (or NMP/MeOH 3:1) and DTT (0.1–0.2 eq) was added, to allow cyclization to BTH and AP-BTH amino acid/peptide derivatives for 1–3 h at rt (completeness of cyclization was monitored using HPLC analysis). Then, MeOH was concentrated and the resulting solution was extracted with water and EtOAc. The two phases were separated, and the aqueous phase was washed once more with EtOAc. The combined organic phases were washed twice with water and then dried with magnesium sulfate (MgSO_4_). The filtrates were condensed and the oily product that was formed was washed with either DEE or a mixture of DEE/Hex (or Hex), and the product was dried in vacuo.

##### 3.3.4.2. Cleavage/Cyclization into Side-Chain *^t^*Bu-Deprotected/Fmoc-Deprotected BTH and AP-BTH Amino Acids and Peptides (**3a**, **3b**, **3c**, **3d**, **3e**, **3f**, **3g**, **34**, **35**, **36**, **37**, **38**, **41**, **43**)

Resin-bound *^t^*Bu-protected/Fmoc-deprotected derivatives were treated with TFA/DCM/TES (90:5:5) for 15 min at rt, the cleavage mixture was filtered, and the resin was washed twice with a fresh cleavage mixture. The combined filtrates were kept under stirring for 1–3 h to allow full deprotection of the side-chain protecting groups and then concentrated on a rotary evaporator. Then, depending on the solubility of the products in organic solvents/aqueous phase, two methods were applied:

(a) The oily products (fully deprotected derivatives) were dissolved in MeOH and DTT (0.1–0.2 eq) was added, and the mixture was stirred for 1–3 h at rt to allow cyclization into BTH and AP-BTH amino acid/peptide derivatives (completeness of cyclization was monitored by HPLC analysis). Then, MeOH was removed and the oily product that was formed was washed with either DEE or a mixture of DEE/Hex (or Hex), and the product was dried in vacuo.
(b) The oily products (fully deprotected derivatives) were dissolved in NMP/MeOH (2:1; 3:1) and DTT (0.1–0.2 eq) was added to allow cyclization into BTH and AP-BTH amino acid/peptide derivatives for 1–3 h at rt (completeness of cyclization was monitored using HPLC analysis). MeOH was then concentrated with a flash of nitrogen and the resulting solution was extracted with water and EtOAc. The two phases were separated, and the aqueous phase was washed twice with EtOAc. TLC analysis showed that all BTH and AP-BTH amino acids/peptides tested were collected in the aqueous phase, which was finally lyophilized to afford BTH/AP-BTH amino acid/peptides.

#### 3.3.5. Synthesis of BTH-Amino Acid Library (**9**, **13**, **14**, **15**, **16**, **16a**, **17**)

A mixture of acetic acid (AcOH) (0.1 mmol; 5.72 μL), Fmoc-Gly-OH (0.1 mmol; 29.73 mg), Fmoc-L-Ala-OH (0.1 mmol, 38.24 mg), Fmoc-L-Lys(Boc)-OH (0.1 mmol; 46.85 mg), Fmoc-L-Cys(Trt)-OH (0.1 mmol; 58.57 gr) and Fmoc-L-Ser(*^t^*Bu)-OH (0.1 mmol; 38.34 mg) were dissolved in NMP (6 mL/gr resin; 1.2 mL) and the resulting mixture of reactants was cooled at 4 °C. Then, DIC (0.66 mmol; 103.34 μL) was added and the mixture was vortexed for 1 min and added to the pre-swollen resin **7** (0.5 mmol/gr; 0.1 mmol available amine groups; 0.2 g). The resin was agitated gently for 3 h at rt and then washed with NMP (×5), iPrOH (×3), and DEE (×2) and dried in vacuo. Cleavage and subsequent cyclization from the resin with protocol 3.3.4.1 resulted in the formation of the BTH-AAs library, which was directly subjected to HPLC analysis (crude mixture).

#### 3.3.6. Scale-Up Protocols

##### Synthesis of BTH-Peptides **18**, **18a** and **18b**

Resin **7** (0.5 mmol/g resin; 0.25 mmol; 0.5 g) was subjected to SPS using Fmoc-Gly-OH (0.75 mmol; 0.223 g), Fmoc-L-Glu(*^t^*Bu)-OH (0.75 mmol; 0.319 g), Fmoc-L-Ala-OH (1.5 mmol; 0.234 g), and Fmoc-L-Asp(*^t^*Bu)-OH (1.5 mmol; 0.309 g) using protocol 3.3.3.1a for the coupling of the first amino acid, and then protocol 3.3.3.3 for the peptide assembly. Fmoc removal during peptide synthesis and at the end of the synthesis was accomplished using protocol 3.3.3.4. The activation of the first Fmoc-amino acid (Fmoc-Gly-OH) during the coupling reaction with resin **7** was performed using DIC (0.825 mmol; 129.17 μL) as the activating agent. This step was performed twice and resulted in complete coupling of the free amine groups, and therefore no capping of any unreacted amine groups in resin **7** was necessary. The next couplings (with Fmoc-L-Glu(*^t^*Bu)-OH, Fmoc-L-Ala-OH, Fmoc-L-Asp(*^t^*Bu)-OH) were performed using HOBt (1.125 mmol; 0.152 g) and DIC (0.9 mmol; 140.92 μL) as a mixture of activating agents. At the end of the peptide assembly (before the final Fmoc deprotection) the resin was washed with NMP (×5), iPrOH (×3), and DEE (×2) and dried in vacuo and the resin was weighted to have a mass of 0.73 g, indicating a mass increment of 0.23 g (which corresponds to a resin substitution of 0.445 mmol peptide/g resin based on mass increment, in good agreement with the initially calculated substitution of 2-aminobenzenethiol in resin **7**). The purity of synthesis was evaluated by taking a small sample of the resin, which was cleaved through protocols 3.3.4.1.a and 3.3.4.1.b to afford the side-chain *^t^*Bu-protected/Fmoc-protected BTH-peptide **18**, which was subjected to HPLC analysis (purity >96% according to HPLC analysis; 265 nm; Figure S16A). Then, the resin was treated with 25% piperidine (protocol 3.3.3.4) to remove the *N*-terminal Fmoc group and the resin was washed with NMP (×5), iPrOH (×3), and DEE (×2) and dried in vacuo. To isolate **18a**, the resin was initially treated with 0.1% TFA in DCM (these filtrates were discarded) and then treated with 1.1% TFA in DCM/TES (95:5) for 15 min at rt (twice), and the filtrates were collected and concentrated until an oily product was formed. This was dissolved in MeOH and DTT (0.2 eq; 0.1 mmol; 15.4 mg) was added and the mixture was stirred for 3 h at rt. Then, MeOH was concentrated, and DEE was added to the oily product to afford a white solid which was further washed with DEE (×3) to finally obtain **18a** as a white solid (Figure S16B). Finally, **18a** was further treated with TFA/DCM/TES 90/5/5 for 1 h and then concentrated to an oily residue. To this, DEE was added and the resulting solid was washed with DEE (×3) to finally obtain **18b** as a white solid in high purity (>97%; 254 nm; Figure S16C). Total yield of **18b**: (93.52 mg; 78%).

##### Synthesis of 2-(4-Aminophenyl)benzothiazole (4-AP-BTH) (**31a**) and 2-(3-Aminophenyl)benzothiazole (3-AP-BTH) (**31b**)

Resin **30** (0.5 mmol/g resin; 0.05 mmol; 100 mg) was treated with TFA/DCM/TES 10/85/5 for 10 min at rt and the cleavage mixture was filtered and further washed twice with a fresh cleavage mixture. The combined filtrates were concentrated and the oily product that was formed was extracted using a 10% Na_2_CO_3_ solution and DEE. The organic phase was separated and washed with water (three times) and dried. This was further concentrated into an oily residue, which was treated with MeOH/H_2_O (1:1) to afford 4-AP-BTH and 3-AP-BTH as white (light-yellow) solids. Yield: **31a** (10.2 mg; 90%); **31b** (10.7 mg; 95%); **31a**: ^1^H-NMR *δ* (600 MHz, MeOH-*d*_4_) 6.75 (d, *J* = 8.6 Hz, 2H), 7.31–7.37 (m, 1H), 7.44–7.49 (m, 1H), 7.77–7.83 (m, 2H), 7.87–7.92 (m, 2H); ^13^C-NMR *δ* (150 MHz, MeOH-*d*_4_) 114.01, 121.22, 121.25, 121.42, 124.31, 125.98, 128.64, 133.89, 151.86, 153.63, 169.87; ESI-MS *m*/*z* [M + H^+^] found: 227.12; **31b**: ^1^H-NMR *δ* (600 MHz, MeOH-*d*_4_) 6.87 (dd, *J* = 8.0, 1.4 Hz, 1H), 7.24 (t, *J* = 7.8 Hz, 1H), 7.36 (d, *J* = 7.6 Hz, 1H), 7.39–7.45 (m, 2H), 7.48–7.58 (m, 1H), 7.98 (dd, *J* = 8.0, 4.6 Hz, 2H); ^13^C-NMR *δ* (150 MHz, MeOH-*d*_4_) 169.53, 153.54, 148.67, 134.57, 133.77, 129.52, 126.20, 125.09, 122.13, 121.48, 117.75, 116.44, 113.07; ESI-MS *m*/*z* [M + H^+^] found: 227.08.

##### Synthesis of AP-BTH Peptide **43**

Resin **7** (0.5 mmol/g resin; 0.25 mmol; 0.5 g) was suspended in a minimum amount of DCM (the resin was pre-treated with DCM, and then DCM was removed through filtration up to the upper level of the resin) and DIPEA (0.75 mmole; 0.13 mL) was added. To this, 4-(Fmoc-amino)benzoyl chloride (**21a**) {synthesized as analytically described in protocol 3.3.3.2.b [Fmoc-4-ABA-OH (0.75 mmol; 0.27 g); SOCl_2_ (0.765 mmole; 55.56 μL); and NMP (125 μL)]} in DCM (2.5 mL) were added and the reaction proceeded for 3 h at rt. After that time, the reaction mixture was filtered, and the resin was washed with NMP and further treated with 25% piperidine (protocol 3.3.3.4) to remove the Fmoc group. SPPS followed, using Fmoc-L-Lys(Boc)-OH (0.75 mmol; 0.351 g) and DIC (0.9 mmol; 140.92 μL) using protocol 3.3.3.1.a for the coupling of the first Fmoc-L-Lys(Boc)-OH, while the next two Fmoc-L-Lys(Boc)-OH amino acids were inserted into the peptide chain using protocol 3.3.3.3 (besides DIC, HOBt (1.125 mmol; 0.152 g) was used as an additive during the activation of the next two Fmoc-L-Lys(Boc)-OH). Protocol 3.3.3.4 was used for Fmoc removal during peptide assembly. Finally, the Fmoc-protected peptide was washed with NMP (×5), iPrOH (×3), and DEE (×2) and dried in vacuo. In order to test the purity of the synthesis, a probe of the resin (of the Fmoc-protected tripeptide) was treated with 1.1% TFA in DCM/TES (95:5) (protocol 3.3.4.1.b) to afford **42** after cyclization in MeOH with a catalytic amount of DTT (Figure S34D). Then, in order to obtain **43**, the resin was initially treated with 25% piperidine (using protocol 3.3.3.4 to afford the resin-bound Fmoc-deprotected tripeptide), and then washed with NMP (×5), iPrOH (×3), and DEE (×2) and dried in vacuo. In order to isolate **43**, the resin was directly treated with TFA/DCM/TES (90/5/5) for 3 h at rt to allow simultaneous cleavage from the resin and removal of the side-chain-protecting groups. Then, the cleavage mixture was concentrated and the oily product that was formed was dissolved in MeOH, and DTT (0.2 eq; 0.1 mmol; 15.4 mg) was added and the mixture was stirred for 3 h at rt. After that time, MeOH was concentrated and the oil that was formed was treated as in protocol 3.3.4.2.b to finally obtain **43** as a white solid in high purity (>95%; 320 nm; Figure S34E). Total yield of **43**: 129.20 mg; 84.6%.

## 4. Conclusions

In this work, we describe for the first time the solid-phase synthesis (SPS) of C-terminal modified 2-benzothiazolyl (BTH) (**3**, **4**) and 2-(aminophenyl)benzothiazolyl (AP-BTH) amino acids and peptides (**5**, **6**). The synthesis of **3**, **4** was achieved using resin-bound 2-aminobenzenethiol (**7**), which was efficiently coupled with Fmoc-amino acids using DIC (or HOAt/DIC) as the condensing agent. Removal of the Fmoc group and subsequent cleavage from the resin through acidic treatment and cyclization (in the presence of DTT) resulted in the preparation of C-modified BTH amino acids **3**, whereas, when SPPS methods were applied, the corresponding C-modified BTH peptides **4** were formed. The synthesis of C-terminal modified 2-(3-aminophenyl)benzothiazolyl and 2-(4-aminophenyl)benzothiazolyl (AP-BTH) amino acids **5** and peptides **6** was achieved by initially introducing the 3-aminobenzoic acid and 4-aminobenzoic acid skeleton to resin-bound 2-aminobenzenethiol (**7**). This was easily carried out via the chlorination of Fmoc-3-aminobenzoic acid (**21a**) and Fmoc-4-aminobenzoic acid (**21b**) with SOCl_2_ in the presence of NMP as catalyst, and the coupling of **21a**/**b** with resin **7**. Removal of the Fmoc group and coupling of the first amino acid (using DIC or HOAt/DIC) and SPPS, followed by cleavage from the resin and cyclization, gave the desired AP-BTH amino acids **5** and peptides **6**. The reactions proceeded with considerable purity, while the low degree of racemization that was measured during the coupling of the first Fmoc-amino acid to resin-bound 2-aminobenzenethiol **7** and resin-bound 2-*N*-aminobenzoyl-aminobenzenethiol **30**, reveals the effectiveness of the proposed methods for the synthesis of C-terminal BTH and AP-BTH amino acids and peptides.

## Figures and Tables

**Table 1:** Racemization (% D-isomer) of H-L-AA1-BTH, measured by the formation of the H-L-Ala-L-AA1-BTH and H-L-Ala-D-AA1-BTH diastereomers and HPLC analysis.

AA1	Rac% ^1^	Rac% ^2^	Rac% ^3^	Rac% ^4^
Leu	0.25	0.14		
Ser(*^t^*Bu)	0.25		<0.10	
Arg(Pbf)	0.41		0.32	
Glu(*^t^*Bu)	1.13		0.98	
Lys(Boc)	1.16		0.58	
Cys(Trt)	1.89	2.22	1.32	
His(Trt)	44.9	40.2	7.64	24.9

^1^ Racemization of AA1 when DIC was used as the condensing agent. ^2^ Racemization of AA1 when DIC (slowly added) was used as the condensing agent. ^3^ Racemization of AA1 when HOAt/DIC was used as the condensing agent. ^4^ Racemization of AA1 when pyOxim/DIPEA was used as the condensing agent.

**Table 2:** Racemization (% D-isomer) of H-L-AA1-AP-BTH, measured by the formation of the H-L-Ala-L-AA1-AP-BTH and H-L-Ala-D-AA1-AP-BTH diastereomers and HPLC analysis.

AA1	Rac% ^1^	Rac% ^2^
Arg(Pbf)	0.14	
Glu(*^t^*Bu)	0.23	
Lys(Boc)	<0.10	
Cys(Trt) ^3^	1.08	
His(Trt)	44.5	7.65

^1^ Racemization of AA1 when DIC was used as the condensing agent. ^2^ Racemization of AA1 when HOAt/DIC was used as the condensing agent. ^3^ In this case the tripeptide H-L-Lys-L-Ala-L-Cys-AP-BTH and the corresponding H-L-Lys-L-Ala-D-Cys-AP-BTH were synthesized.

## Data Availability

The data presented in this study are available on request from the corresponding author.

## Supplementary Materials

The following supporting information can be downloaded at: https://www.mdpi.com/article/10.3390/molecules28145412/s1, Figures S1–S14: HPLC/ESI-MS analysis of BTH-AAs (**3a**–**3g**); Figures S15–S17: HPLC/ESI-MS analysis of BTH-peptide library (**9**, **13**, **14**, **15**, **16**, **16a**, **17**) and BTH-peptide derivatives (**18**, **18a**, **18b**, **19**, **20**); Figures S18–S21: ^1^H and ^13^C-NMR of Fmoc-4-ABA-OH (**21a**) and Fmoc-3-ABA-OH (**21b**); Figures S22–S27: HPLC/ESI-MS analysis of synthesized **21a/b**; the corresponding methyl esters (formed by the reaction of chlorinated **21a/b** with MeOH (in absence and presence of NMP); **29a/b** obtained by the reaction of chlorinated **21a/b** with resin **7** and acidic treatment of resin **28** and cyclization; ^1^H and ^13^C-NMR of **31a/b**; Figures S28–S37: HPLC/ESI-MS analysis of AP-BTH-AAs (**34**–**38**); Figure S38: HPLC/ESI-MS analysis of AP-BTH-peptides (**39**–**43**).

## Sample Availability

Samples of all compounds that are described in this paper are available from the authors.
