# Supplementary material for: Solid-Phase Synthesis of 2-Benzothiazolyl and 2-(Aminophenyl)benzothiazolyl Amino Acids and Peptides
Source: Molecules. 2023 Jul 14;28(14):5412. doi: 10.3390/molecules28145412 (PMC10385376; doi:10.3390/molecules28145412)
Supplement: Supplementary file 1 [file molecules-28-05412-s001.zip › molecules-2492670-supplementary.pdf]

Solid-Phase Synthesis of 2-Benzothiazolyl and 2-(Aminophenyl)benzothiazolyl amino acids and peptides

Spyridon Mourtas,[1,*] Vasileios Athanasopoulos,[1] Dimitrios Gatos[1] and Kleomenis Barlos[2,*]

[1] *Department of Chemistry, University of Patras, 26510 Rio Patras, Greece.*
[2] *CBL-Patras, Patras Industrial Area, Block 1, 25018 Patras, Greece.*
[*] Correspondence: *s.mourtas@upatras.gr*; Tel.: +30-2610-996015; *barlos@cblpatras.gr*; Tel.: +30-2610-647600

SUPPLEMENTARY MATERIAL

A. HPLC/ESI-MS analysis of BTH-AAs (3a-3g)

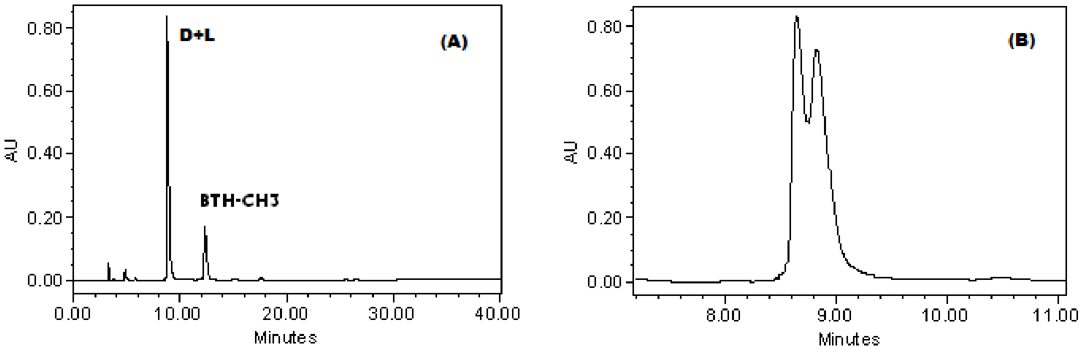

Figure S1. HPLC of the **mixture** (crude) of **3a** (H-L-Ala-L-Leu-BTH) and its **D-isomer** (H-L-Ala-D-Leu-BTH) (synthesized by using DIC as the condensing agent) after cleavage from the resin by treatment with 1.5% TFA in DCM/TES (95:5) (**A**); Enlargement of the area where the two diastereomers elute (**B**); Column: Lichrospher RP-18, 5μm, 125-4 mm; gradient: 20% to 100% AcCN in water (both containing 0.08% TFA) in 30 min; flow rate: 1 mLmL/min; detection at 254 nm.

The eluted peaks were collected and subjected to ESI-MS *m/z*: **3a**: [M+H$^+$] found: 292.40.

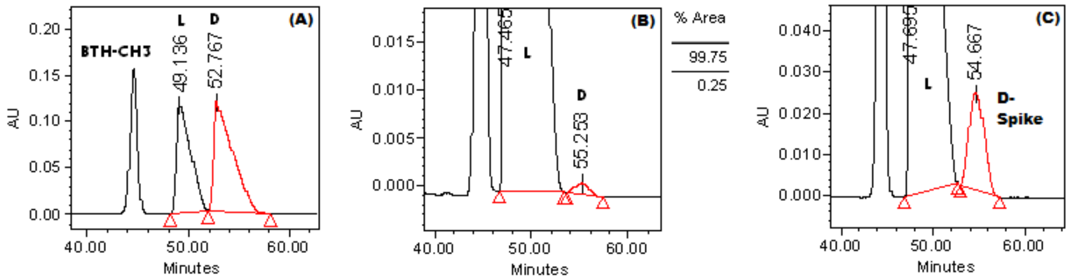

Figure S2. HPLC analysis for determining the enantiomeric purity of the synthesized **3a** (H-L-Ala-L-Leu-BTH): (**A**) Mixture of **3a** (H-L-Ala-L-Leu-BTH) and its H-L-Ala-D-Leu-BTH diastereomer; (**B**) Crude **3a** (synthesized by using DIC as the condensing agent); (**C**) Crude **3a** spiked with its H-L-Ala-D-Leu-BTH diastereomer. Column: YMC-Triart C18, 250x4.6 mmL.D. S-5μm, 12nm; gradient: 22% AcCN (0.08% TFA): 78% water (0.08% TFA) isocratic mixture; flow rate: 1 mLmL/min; detection at 254 nm.

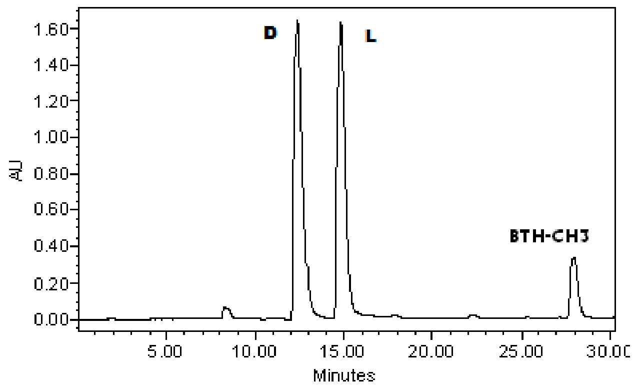

Figure S3. HPLC of the mixture (crude) of **3b** (H-L-Ala-L-Ser-BTH) and its D-isomer (H-L-Ala-D-Ser-BTH) (synthesized by using DIC as the condensing agent) after cleavage from the resin by treatment with TFA/DCM/TES 90/5/5; Column: Lichrospher RP-18, 5μm, 125-4 mm; gradient: 10% to 40% AcCN in water (both containing 0.08% TFA) in 30 min; flow rate: 1 mL/min; detection at 254 nm.

The eluted peaks were collected and subjected to ESI-MS *m/z*: **3b:** [M+H$^+$] found: 266.13.

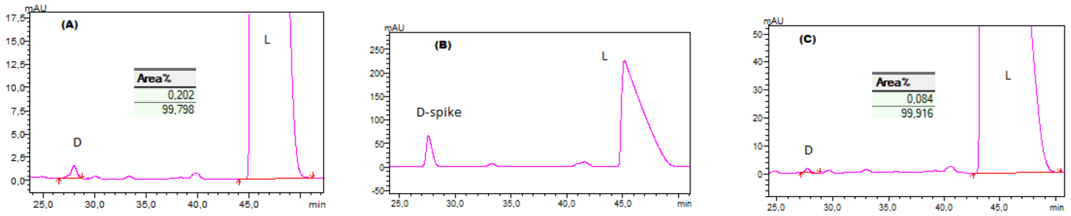

Figure S4. HPLC analysis for determining the enantiomeric purity of **3b** (H-L-Ala-L-Ser-BTH): (**A**) Crude **3b** (synthesized by using DIC as the condensing agent); (**B**) Crude **3b** spiked with its H-L-Ala-D-Ser-BTH diastereomer; (**C**) Crude **3b** (synthesized by HOAt/DIC as the condensing agent); Column: YMC-Triart C18, 250x4.6 mmL.D. S-5µm, 12nm; gradient: 10% AcCN (0.08% TFA): 90% water (0.08% TFA) isocratic mixture; flow rate: 1 mL/min; detection at 254 nm.

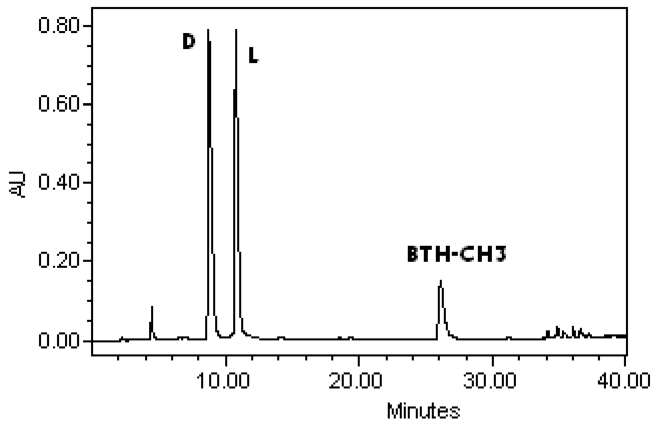

Figure S5. HPLC of the mixture (crude) of **3c** (H-L-Ala-L-Arg-BTH) and its D-isomer (H-L-Ala-D-Arg-BTH) (synthesized by using DIC as the condensing agent) after cleavage from the resin by treatment with TFA/DCM/TES 90/5/5; Column: Lichrospher RP-18, 5μm, 125-4 mm; gradient: 10% to 40% AcCN in water (both containing 0.08% TFA) in 30 min; flow rate: 1 mL/min; detection at 254 nm.

The eluted peaks were collected and subjected to ESI-MS *m/z*:: **3c:** [M+H$^+$] found: 335.42.

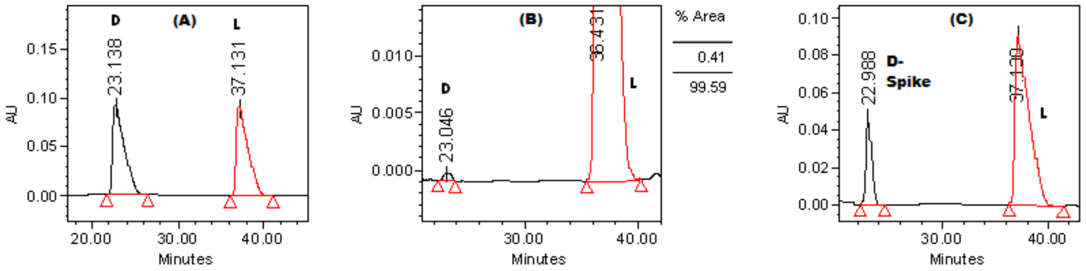

Figure S6. HPLC analysis for determining the enantiomeric purity of the synthesized **3c** (H-L-Ala-L-Arg-BTH): (**A**) Mixture of **3c** (H-L-Ala-L-Arg-BTH) and its H-L-Ala-D-Arg-BTH diastereomer; (**B**) Crude **3c** (synthesized by using DIC as the condensing agent); (**C**) Crude **3c** spiked with its H-L-Ala-D-Arg-BTH diastereomer. Column: YMC-Triart C18, 250x4.6 mmL.D. S-5µm, 12nm; gradient: 12% AcCN (0.08% TFA): 88% water (0.08% TFA) isocratic mixture; flow rate: 1 mL/min; detection at 254 nm.

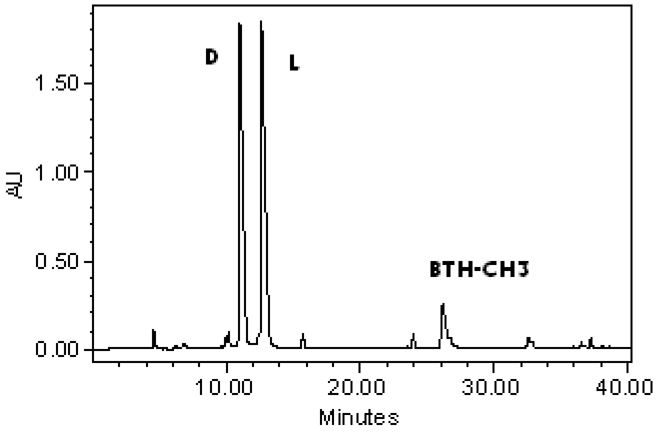

Figure S7. HPLC of the mixture (crude) of **3d** (H-L-Ala-L-Glu-BTH) and its D-isomer (H-L-Ala-D-Glu-BTH) (synthesized by using DIC as the condensing agent) after cleavage from the resin by treatment with TFA/DCM/TES 90/5/5; Column: Lichrospher RP-18, 5μm, 125-4 mm; gradient: 10% to 40% AcCN in water (both containing 0.08% TFA) in 30 min; flow rate: 1 mL/min; detection at 254 nm.

The eluted peaks were collected and subjected to ESI-MS *m/z*: **3d:** [M+H$^+$] found: 307.90.

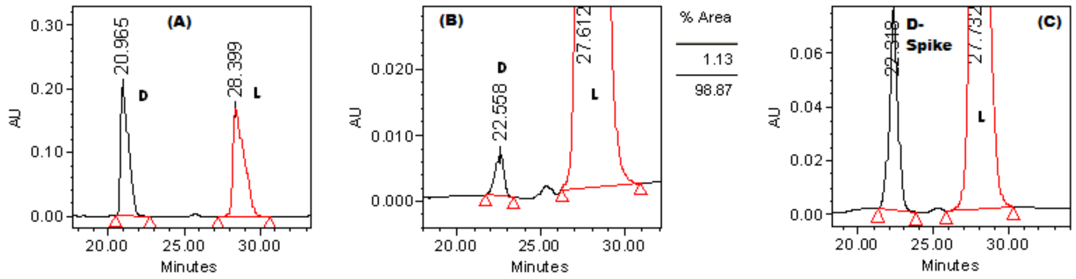

Figure S8. HPLC analysis for determining the enantiomeric purity of the synthesized **3d** (H-L-Ala-L-Glu-BTH): (**A**) Mixture of **3d** (H-L-Ala-L-Glu-BTH) and its H-L-Ala-D-Glu-BTH diastereomer; (**B**) Crude **3d** (synthesized by using DIC as the condensing agent); (**C**) Crude **3d** spiked with its H-L-Ala-D-Glu-BTH diastereomer. Column: Lichrospher RP-18, 5μm, 125-4 mm; gradient: 10% to 14% AcCN in water (both containing 0.08% TFA) in 30 min; flow rate: 1 mL/min; detection at 254 nm.

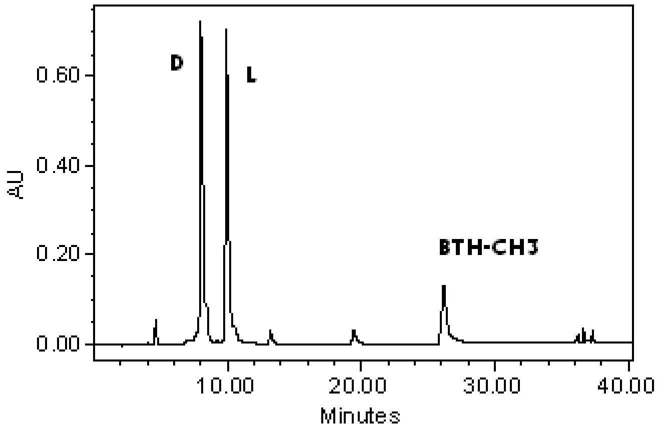

Figure S9. HPLC of the mixture (crude) of **3e** (H-L-Ala-L-Lys-BTH) and its D-isomer (H-L-Ala-D-Lys-BTH) (synthesized by using DIC as the condensing agent) after cleavage from the resin by treatment with TFA/DCM/TES 90/5/5; Column: Lichrospher RP-18, 5μm, 125-4 mm; gradient: 10% to 40% AcCN in water (both containing 0.08% TFA) in 30 min; flow rate: 1 mL/min; detection at 254 nm.

The eluted peaks were collected and subjected to ESI-MS *m/z*: **3e:** [M+H$^+$] found: 307.27.

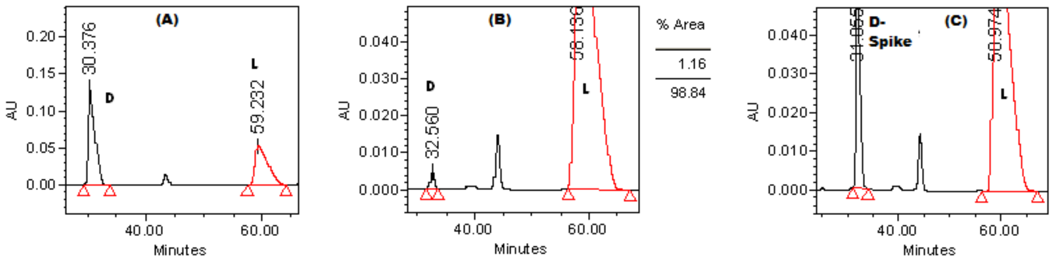

Figure S10. HPLC analysis for determining the enantiomeric purity of the synthesized **3e** (H-L-Ala-L-Lys-BTH): (**A**) Mixture of **3e** (H-L-Ala-L-Lys-BTH) and its H-L-Ala-D-Lys-BTH diastereomer; (**B**) Crude **3e** (synthesized by using DIC as the condensing agent); (**C**) Crude **3e** spiked with its H-L-Ala-D-Lys-BTH diastereomer. Column: YMC-Triart C18, 250x4.6 mmL.D. S-5µm, 12nm; gradient: 10% AcCN (0.08% TFA): 90% water (0.08% TFA) isocratic mixture; flow rate: 1 mL/min; detection at 254 nm.

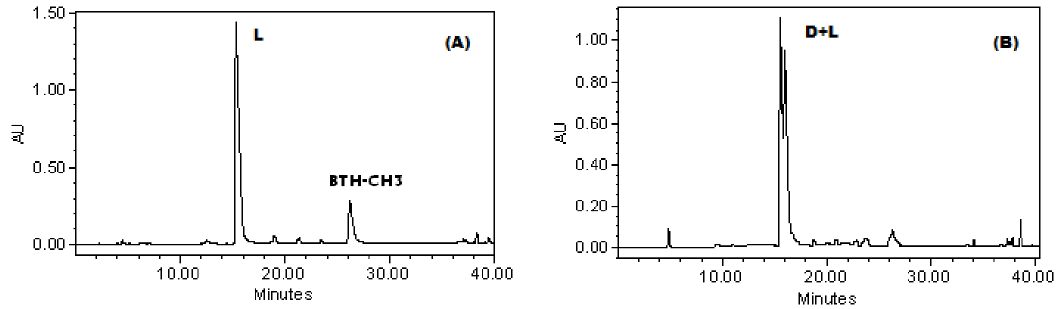

Figure S11. HPLC of crude **3f** (H-L-Ala-L-Cys-BTH) (**A**) and the mixture of **3f** with its D-isomer (H-L-Ala-D-Cys-BTH) (**B**) (synthesized by using DIC as the condensing agent) after cleavage from the resin by treatment with TFA/DCM/TES 90/5/5; Column: Lichrospher RP-18, 5μm, 125-4 mm; gradient: 10% to 40% AcCN in water (both containing 0.08% TFA) in 30 min; flow rate: 1 mL/min; detection at 254 nm.

The eluted peaks were collected and subjected to ESI-MS *m/z*: **3f:** [M+H$^+$] found: 281.90.

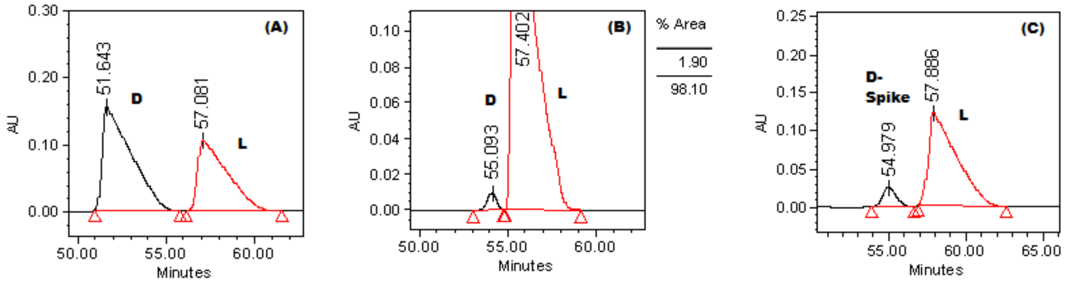

Figure S12. HPLC analysis for determining the enantiomeric purity of the synthesized **3f** (H-L-Ala-L-Cys-BTH): (**A**) Mixture of **3f** (H-L-Ala-L-Cys-BTH) and its H-L-Ala-D-Cys-BTH diastereomer; (**B**) Crude **3f** (synthesized by using DIC as the condensing agent); (**C**) Crude **3f** spiked with its H-L-Ala-D-Cys-BTH diastereomer. Column: YMC-Triart C18, 250x4.6 mmL.D. S-5μm, 12nm; gradient: 15% AcCN (0.08% TFA): 85% water (0.08% TFA) isocratic mixture; flow rate: 1 mL/min; detection at 254 nm.

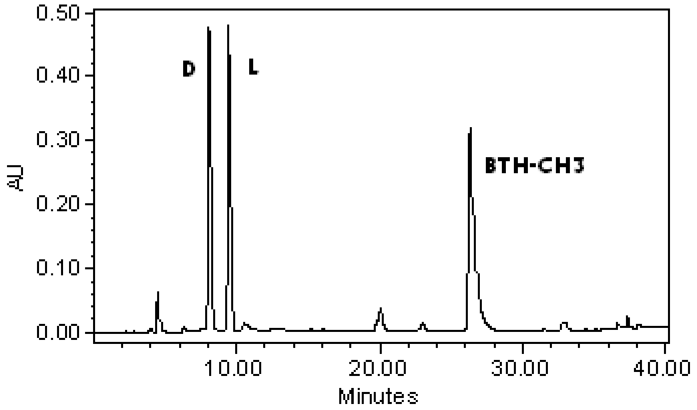

Figure S13. HPLC of the mixture (crude) of **3g** (H-L-Ala-L-His-BTH) and its D-isomer (H-L-Ala-D-His-BTH) (synthesized by using DIC as the condensing agent) after cleavage from the resin by treatment with TFA/DCM/TES 90/5/5; Column: Lichrospher RP-18, 5*μ*m, 125-4 mm; gradient: 10% to 40% AcCN in water (both containing 0.08% TFA) in 30 min; flow rate: 1 mL/min; detection at 254 nm.

The eluted peaks were collected and subjected to ESI-MS *m/z*: **3g:** [M+H$^+$] found: 317.56.

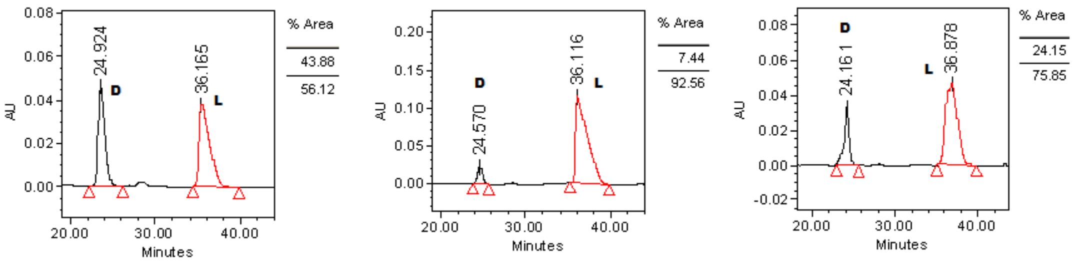

Figure S14. HPLC analysis for determining the enantiomeric purity of the synthesized **3g** (H-L-Ala-L-His-BTH): (**A**) Crude **3g** (synthesized by using DIC as the condensing agent); (**B**) Crude **3g** (synthesized by using HOBt/DIC as the condensing agent); (**C**) Crude **3g** (synthesized by using pyOxim/DIPEA as the condensing agent). Column: YMC-Triart C18, 250x4.6 mmL.D. S-5µm, 12nm; gradient: 10% AcCN (0.08% TFA): 90% water (0.08% TFA) isocratic mixture; flow rate: 1 mL/min; detection at 254 nm.

B. HPLC/ESI-MS analysis of BTH-peptide library (9, 13, 14, 15, 16, 16a, 17) and BTH-peptide derivatives (18, 18a, 18b, 19, 20)

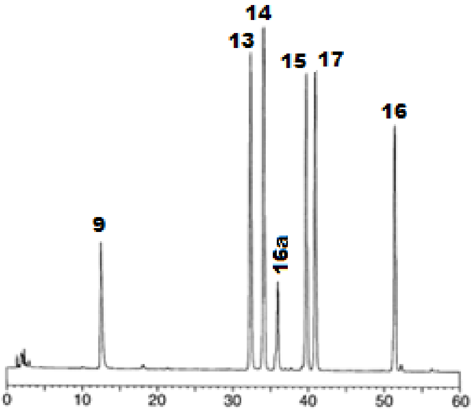

Figure S15. HPLC analysis of the crude BTH amino acid library **9**, **13**, **14**, **15**, **16**, **16a**, **17** (after cleavage and treatment with protocol 1.3.4.1); Column: Lichrospher RP-8, 5 μm, 125-4 mm; gradient: 20% to 90% AcCN in water in 60 min; flow rate: 1 mL/min; detection at 265 nm.

The eluted peaks were collected and subjected to ESI-MS *m/z*: **9**: [M+H$^+$] found:150.16; **13**: [M+H$^+$] found: 388.02; **14**: [M+H$^+$] found: 401.46; **15**: [M+H$^+$] found: 558.34; **16**: [M+H$^+$] found: 675.14; **16a**: [M+H$^+$] found: 433.40; **17**: [M+H$^+$] found: 473.40.

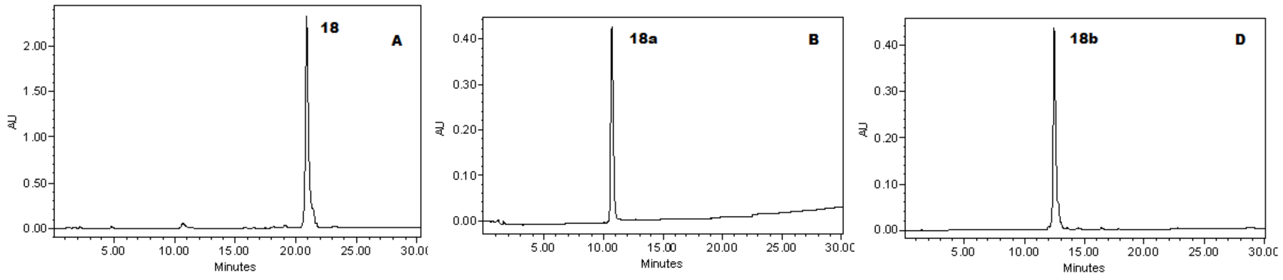

Figure S16. HPLC analysis of **18** (A), **18a** (B), **18b** (C, D); Column: Lichrospher RP-18, 5μm, 125-4 mm. (**A**) **18**; gradient: 20% to 100% AcCN in water in 30 min; flow rate: 1 mL/min; detection at 265 nm. (**B**) **18a**; gradient: 20% to 100% AcCN in water in 30 min; flow rate: 1 mL/min; detection at 254 nm. (**C**) **18b**; gradient: 0% to 60% AcCN in water in 30 min; flow rate: 1 mL/min; detection at 254 nm.

The eluted peaks were collected and subjected to ESI-MS *m/z*: **18**: [M+H$^+$] found: 814.63; **18a**: [M+H$^+$] found: 592.31; **18b**: [M+H$^+$] found: 480.40.

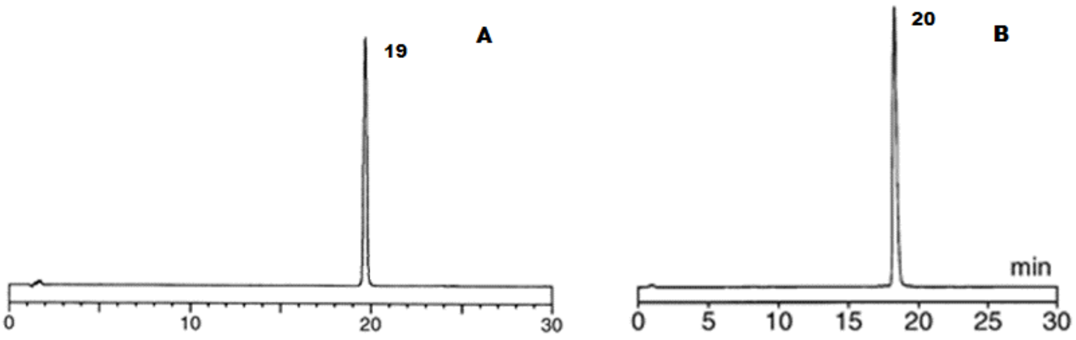

Figure S17. (**A**) HPLC analysis of **19** after cleavage and treatment with method 1.3.4.1-a; Column: Lichrosphere RP-8, 5 μm, 125-4 mm; gradient: 50 to 100% AcCN in water in 30 min; flow rate: 1 mL/min; detection at 265 nm; (**B**) HPLC analysis of **20** after cleavage and treatment with method 1.3.4.1-a; Column: Zorbax SB-C18, 3.5 μm, 30-2.1 mm; gradient: 50 to 100% AcCN in water in 20 min; flow rate: 0.4 mL/min; detection at 265 nm.

The eluted peaks were collected and subjected to ESI-MS *m/z*: **19**: [M+H$^+$] found: 1444.80; **20**: [M+H$^+$] found: 1699.23.

C. ^{1}H and ^{13}C-NMR of Fmoc-4-ABA-OH (21a) and Fmoc-3-ABA-OH (21b)

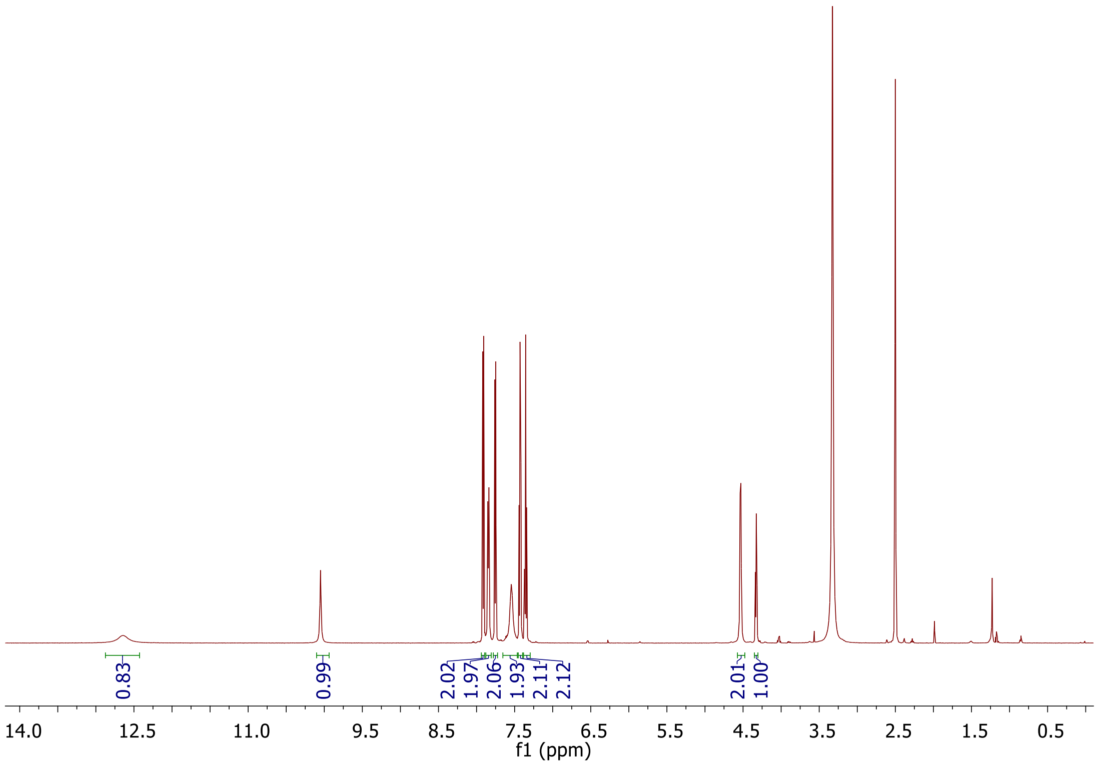

Figure S18: ^{1}H-NMR δ (600 MHz, DMSO-d_6) of Fmoc-4-ABA-OH (**21a**).

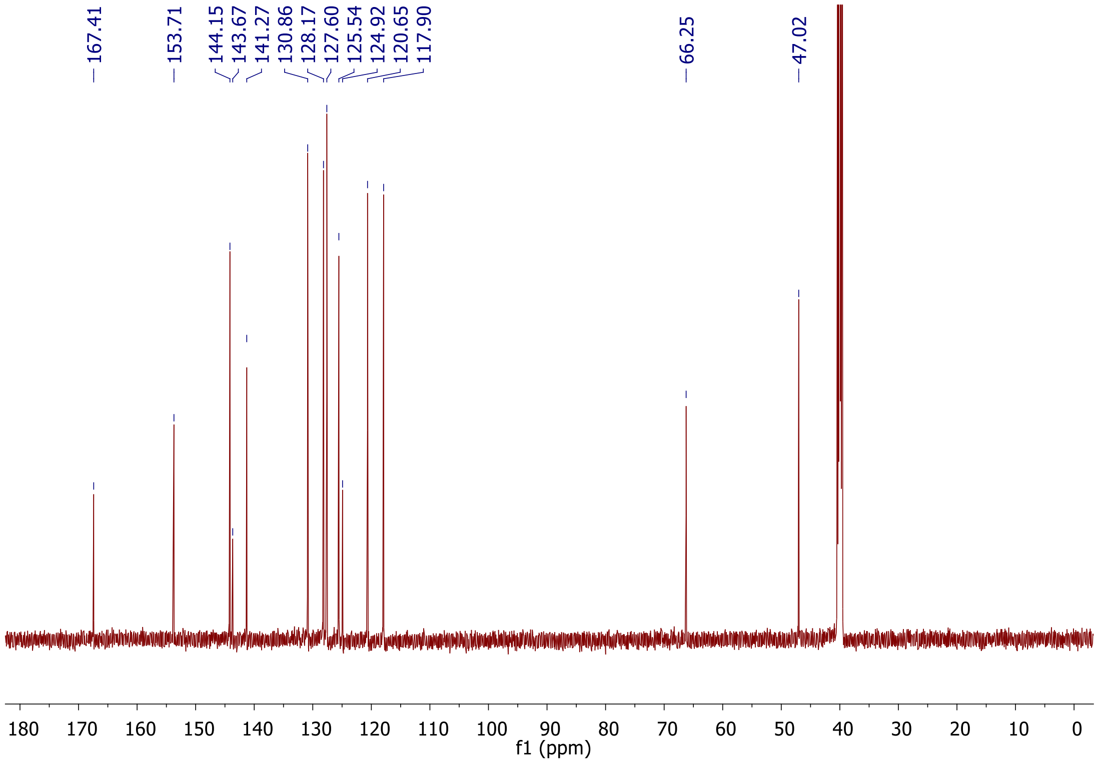

Figure S19: ^{13}C-NMR δ (150 MHz, DMSO-d_6) of Fmoc-4-ABA-OH (**21a**).

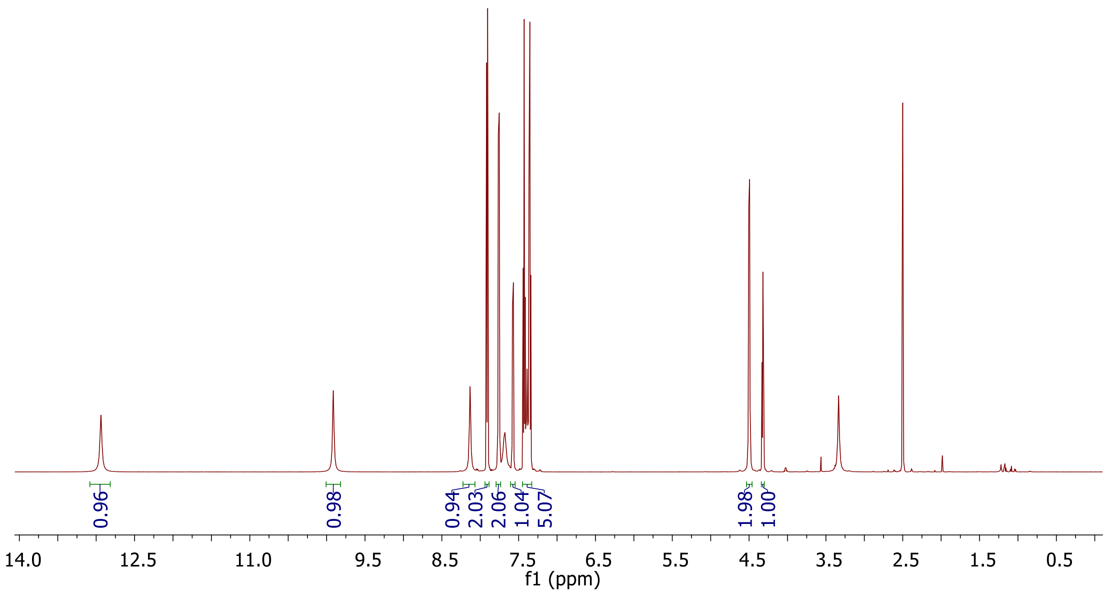

Figure S20: ^{1}H-NMR δ (600 MHz, DMSO-d_6) of Fmoc-3-ABA-OH (**21b**).

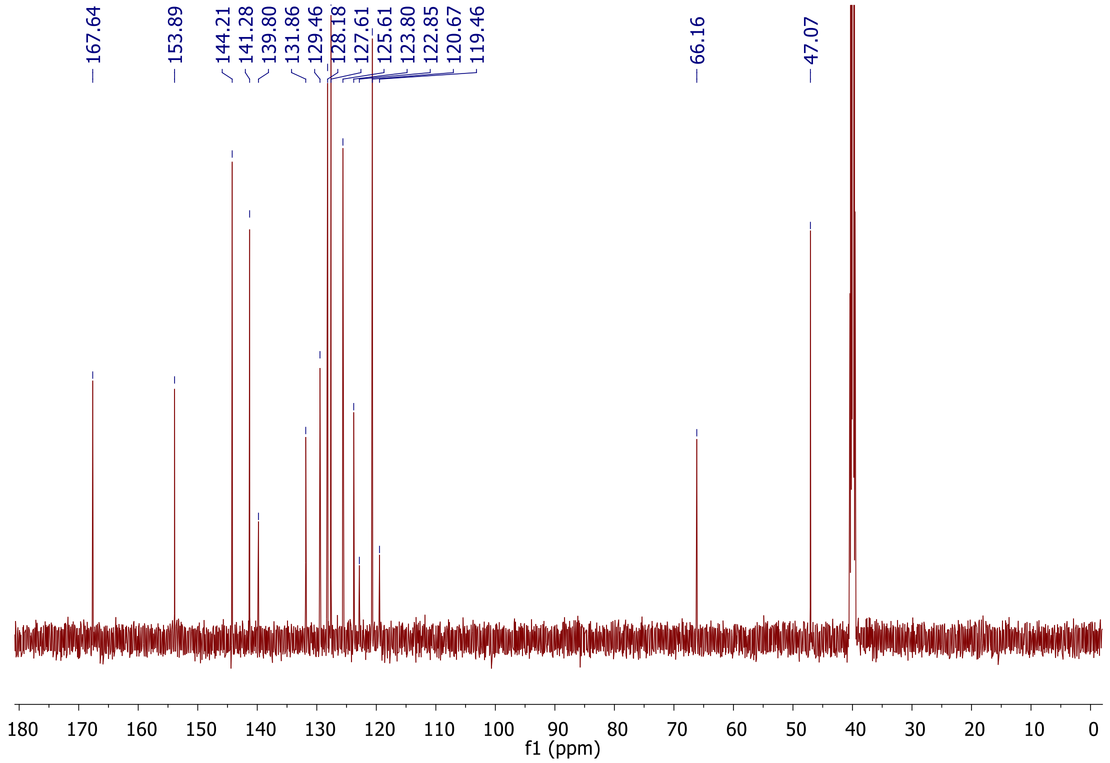

Figure S21: ^{13}C-NMR δ (150 MHz, DMSO-d_6) of Fmoc-3-ABA-OH (**21b**).

D. HPLC/ESI-MS analysis of synthesized 21a/b; the corresponding methyl esters (formed by the reaction of chlorinated 21a/b with MeOH (in absence and presence of NMP); 29a/b obtained by the reaction of chlorinated 21a/b with resin 7 and acidic treatment of resin 28 and cyclization; ^{1}H and ^{13}C-NMR of 31a/b

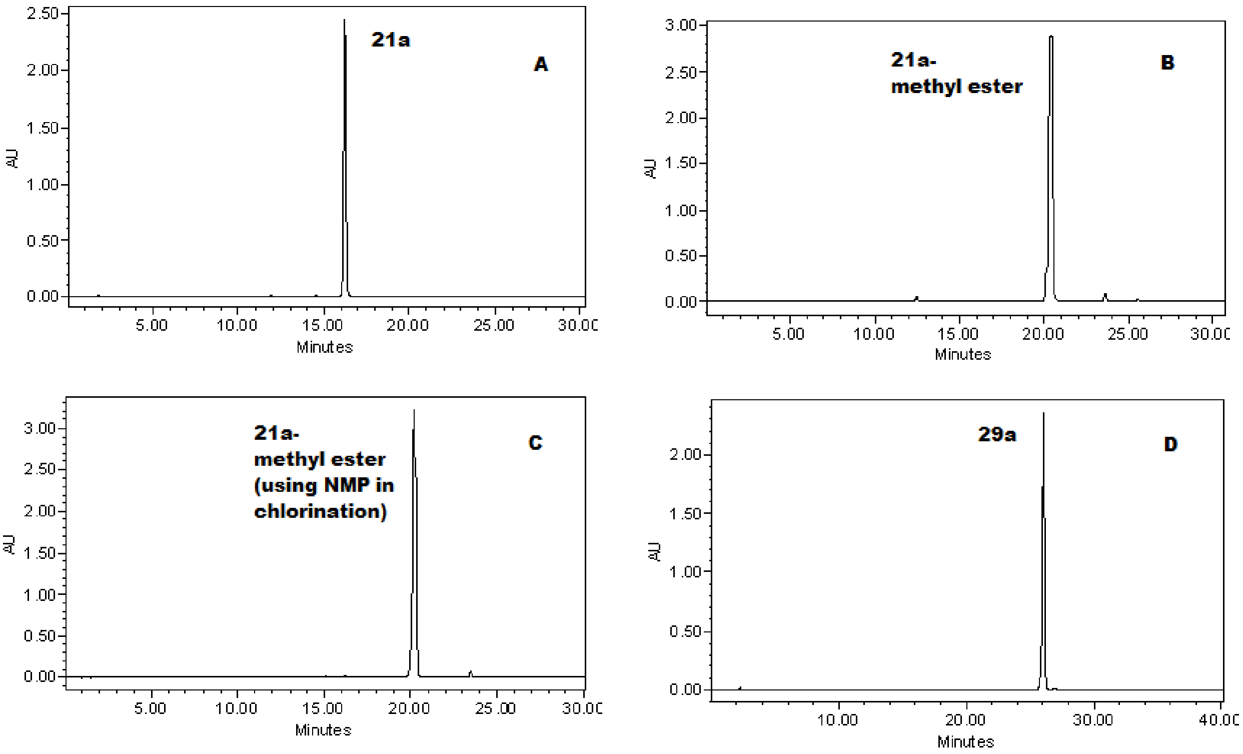

Figure S22. Representative HPLC profile of: **21a** (265nm) (**A**); the methyl ester of **21a** formed by quenching with MeOH a small sample of chlorinated **21a** (**22a**) with $SOCl_2$ (overnight reaction) (265nm) (**B**); the methyl ester of **21a** formed by quenching with MeOH a small sample of activated **21a** in $SOCl_2$/NMP (265nm) (**C**); **29a** synthesized by the reaction of activated **21a** (with $SOCl_2$/NMP) with resin **7** (298nm) (**D**); Column: Lichrospher RP-18, 5μm, 125-4 mm; gradient: 20% to 100% AcCN in water in 30 min; flow rate: 1 mL/min; detection at 265 nm.

The eluted peaks were collected and subjected to ESI-MS *m/z*: **21a**: [M+H$^+$] found: 360.38; **21a-OMe**: [M+H$^+$] found: 374.52; **29a**: [M+H$^+$] found: 448.09.

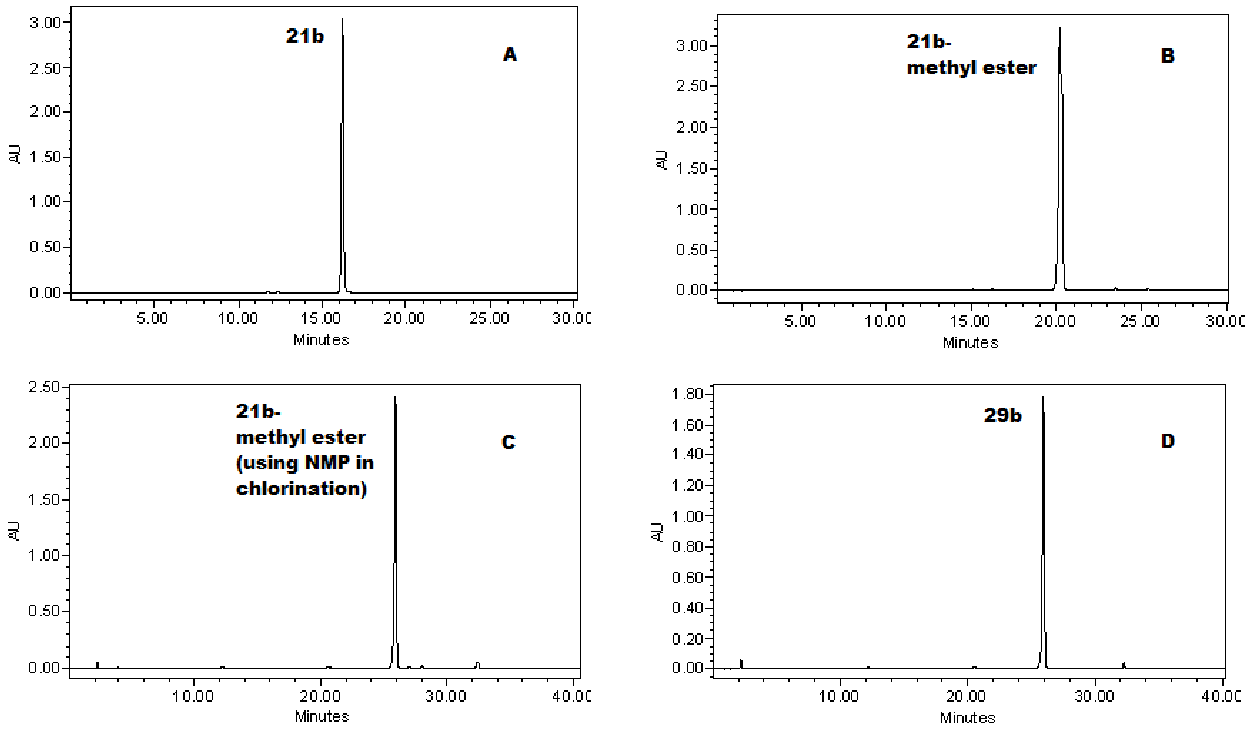

Figure S23. Representative HPLC profile of: **21b** (265nm) (**A**); the methyl ester of **21b** formed by quenching with MeOH a small sample of chlorinated **21b** (**22b**) with $SOCl_2$ (overnight reaction)] (265nm) (**B**); **29b** synthesized by the reaction of chlorinated **21b** (with $SOCl_2$) with resin **7** (298nm) (**C**); **29b** synthesized by the reaction of activated **21b** (with $SOCl_2$/NMP) with resin **7** (298nm) (**D**); Column: Lichrospher RP-18, 5μm, 125-4 mm; gradient: 20% to 100% AcCN in water in 30 min; flow rate: 1 mL/min.

The eluted peaks were collected and subjected to ESI-MS *m/z*: **21b**: [M+H$^+$] found: 360.24; **21b-OMe**: [M+H$^+$] found: 374.48; **29b**: [M+H$^+$] found: 449.32.

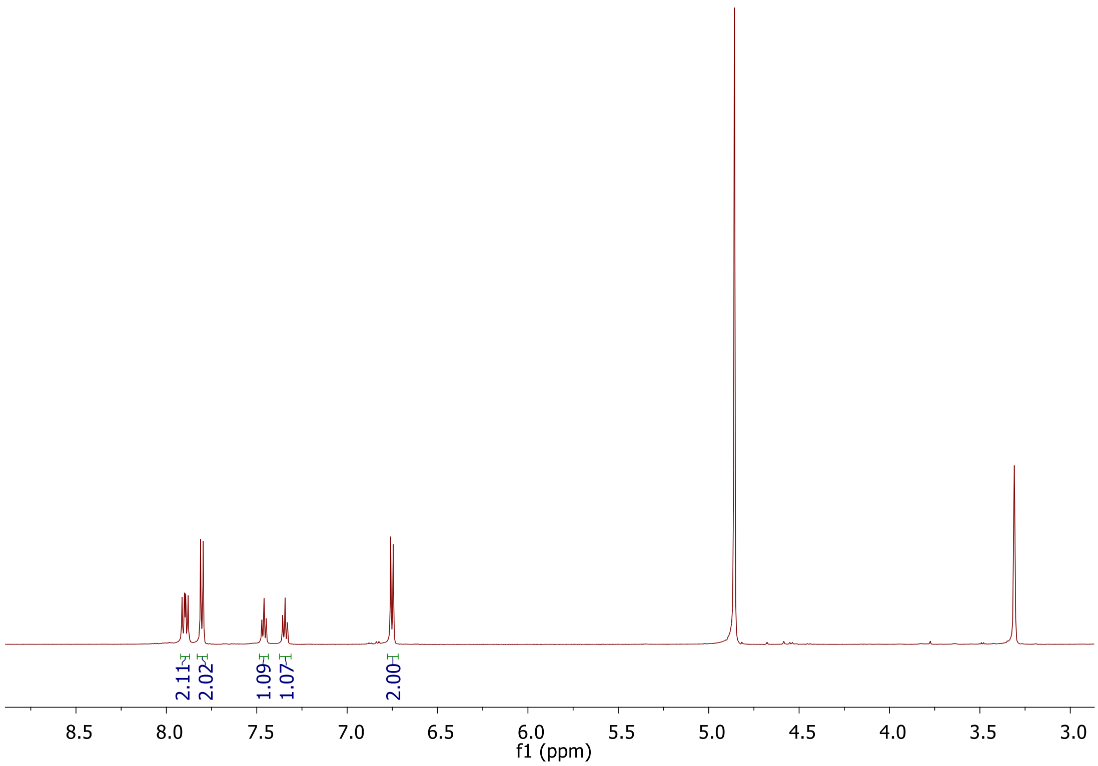

Figure S24. ^{1}H-NMR δ (600 MHz, MeOH-d_4) of 2-(4-aminophenyl)benzothiazole (4-AP-BTH **31a**).

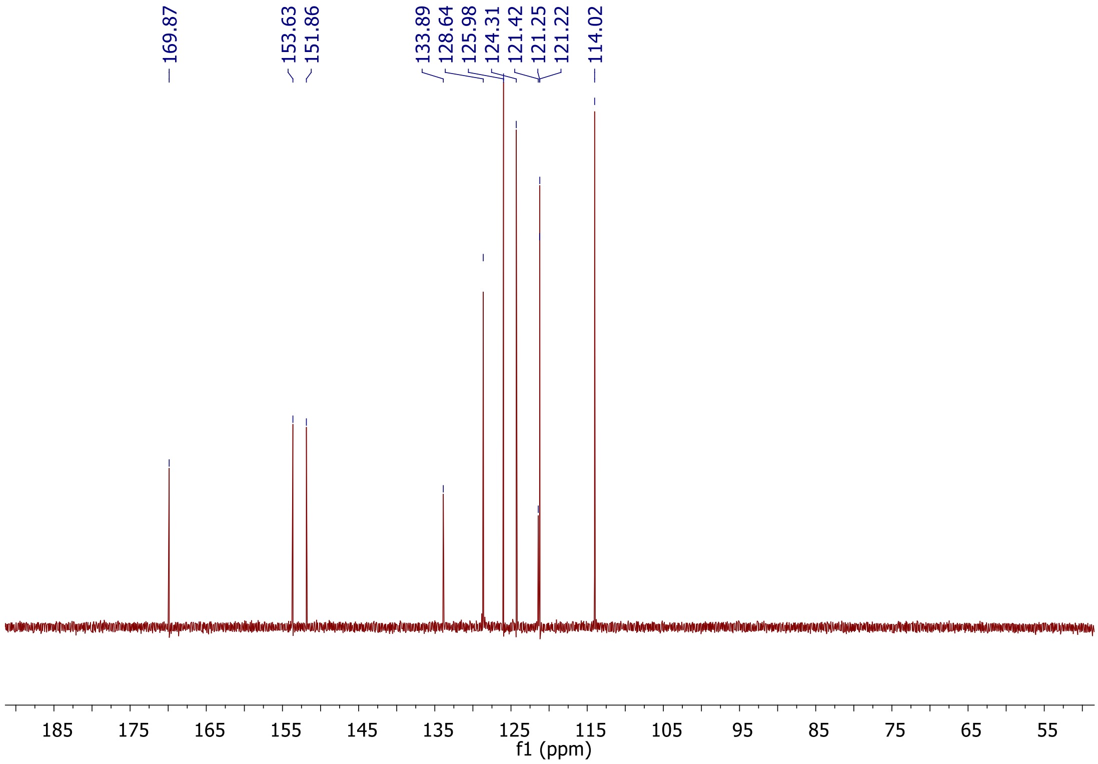

Figure S25. ^{13}C-NMR δ (150 MHz, MeOH-d_4) of 2-(4-aminophenyl)benzothiazole (4-AP-BTH **31a**).

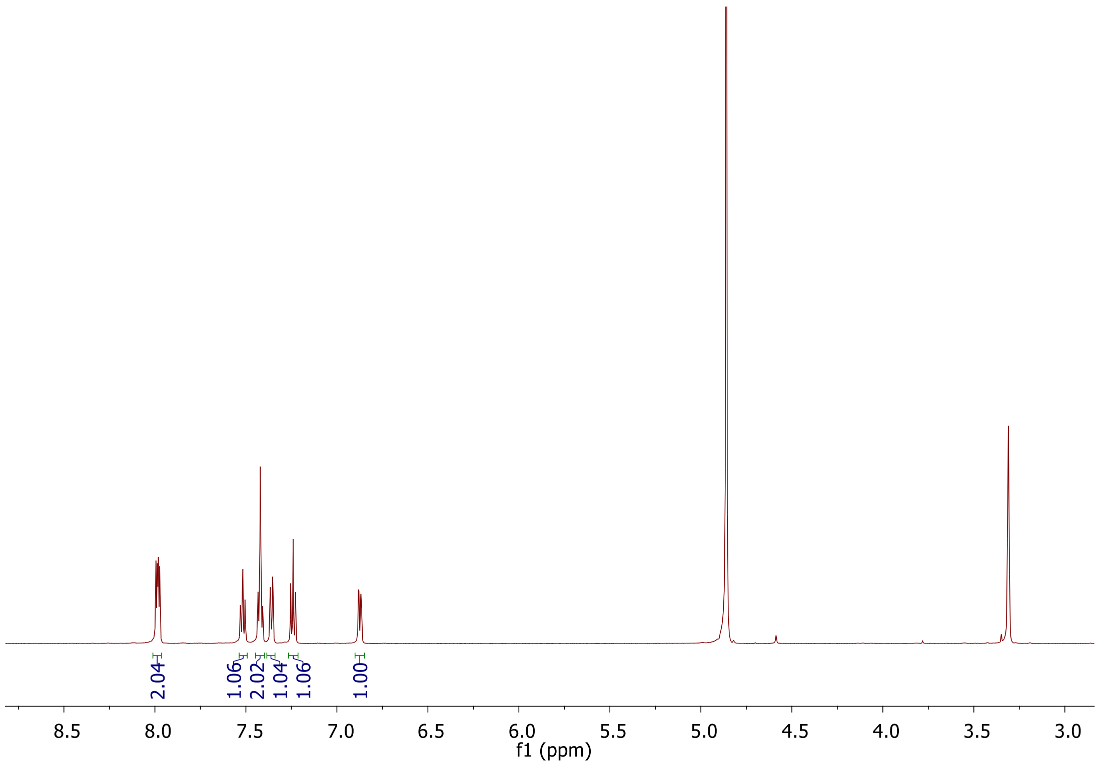

Figure S26. ^{1}H-NMR δ (600 MHz, MeOH-d_4) of 2-(3-aminophenyl)benzothiazole (3-AP-BTH **31b**).

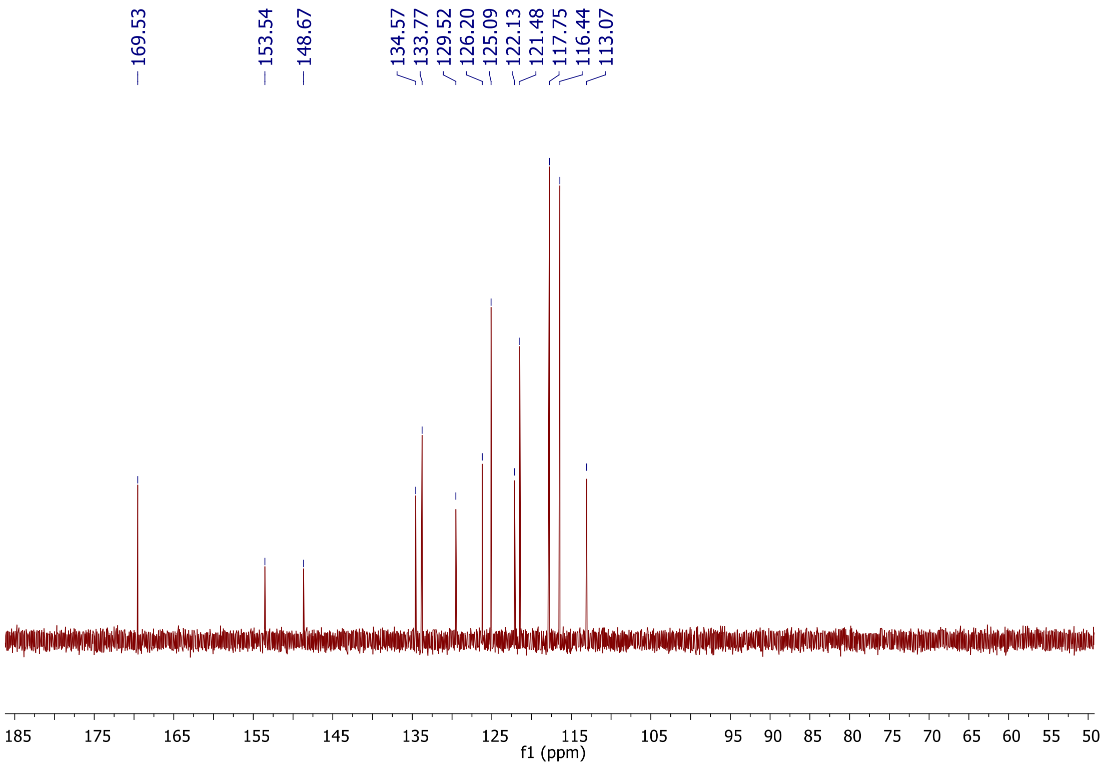

Figure S27. ^{13}C-NMR δ (150 MHz, MeOH-d_4) of 2-(3-aminophenyl)benzothiazole (3-AP-BTH **31b**).

E. HPLC/ESI-MS analysis of AP-BTH-AAs (34-38)

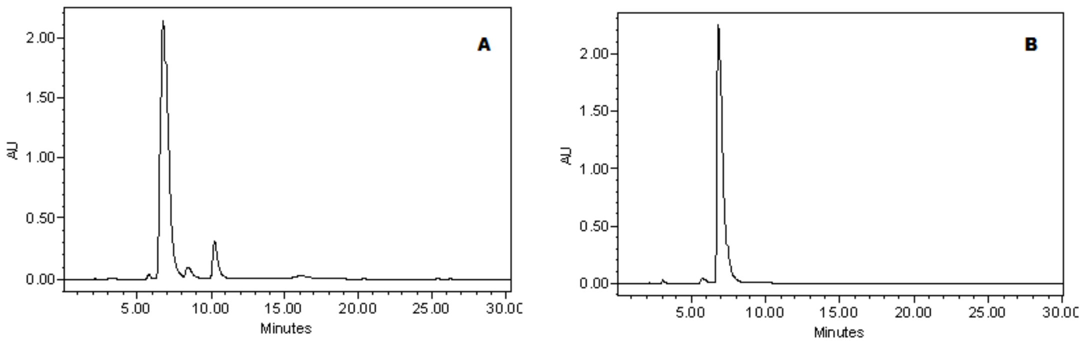

Figure S28. HPLC analysis of **34** (H-L-Ala-L-Arg-AP-BTH) (synthesized by using DIC as the activating agent) after cleavage and treatment using method 1.3.4.3-a (A) and method 1.3.4.3-b (B); Column: Lichrospher RP-18, 5*μ*m, 125-4 mm; gradient: 20% to 100% AcCN in water (both containing 0.08% TFA) in 30 min; flow rate: 1 mL/min; detection at 320 nm.

The eluted peaks were collected and subjected to ESI-MS *m/z*: **34:** [M+H$^+$] found: 454.10.

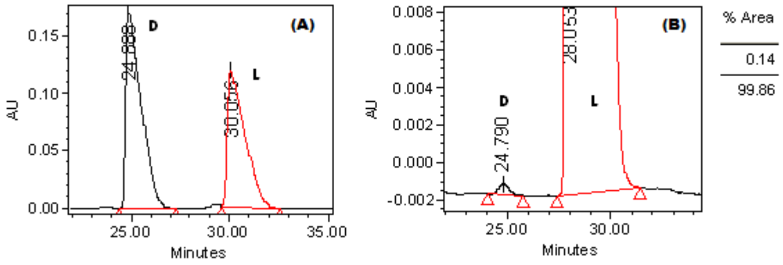

Figure S29. HPLC analysis for determining the enantiomeric purity of the synthesized **34** (H-L-Ala-L-Arg-AP-BTH): (**A**) Mixture of **34** (H-L-Ala-L-Arg-AP-BTH) and its H-L-Ala-D-Arg-AP-BTH diastereomer; (**B**) Crude **34** (synthesized by using DIC as the condensing agent); Column: YMC-Triart C18, 250x4.6 mmL.D. S-5µm, 12nm; gradient: 24% AcCN (0.08% TFA): 76% water (0.08% TFA) isocratic mixture; flow rate: 1 mL/min; detection at 320 nm.

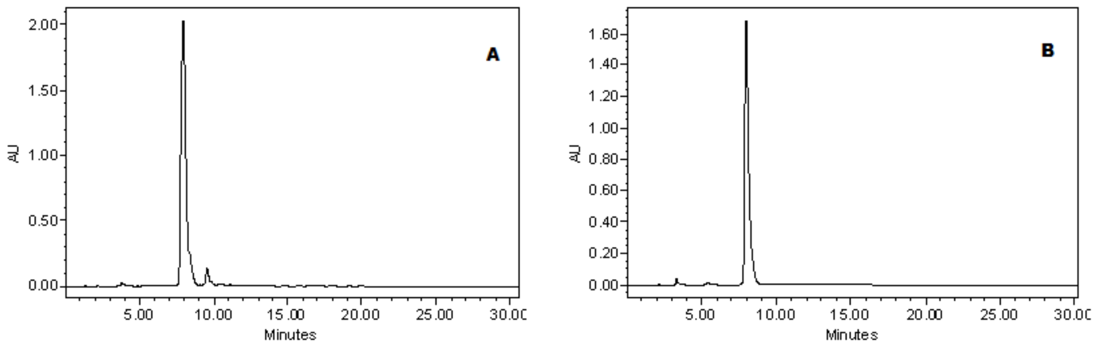

Figure S30. HPLC analysis of **35** (H-L-Ala-L-Glu-AP-BTH) (synthesized by using DIC as the activating agent) after cleavage and treatment using method 1.3.4.3-a (A) and method 1.3.4.3-b (B); Column: Lichrospher RP-18, 5μm, 125-4 mm; gradient: 20% to 100% AcCN in water (both containing 0.08% TFA) in 30 min; flow rate: 1 mL/min; detection at 320 nm.

The eluted peaks were collected and subjected ESI-MS *m/z*: **35:** [M+H$^+$] found: 427.09.

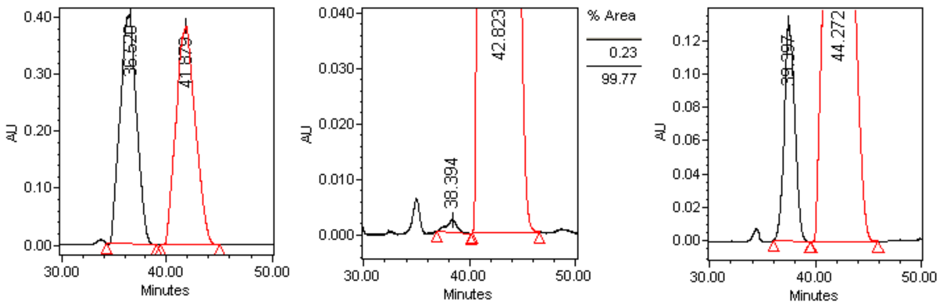

Figure S31. HPLC analysis for determining the enantiomeric purity of the synthesized **35** (H-L-Ala-L-Glu-AP-BTH): (**A**) Mixture of **35** (H-L-Ala-L-Glu-AP-BTH) and its H-L-Ala-D-Glu-AP-BTH diastereomer; (**B**) Crude **35** (synthesized by using DIC as the condensing agent); (**C**) Crude **35** spiked with its D diastereomer (H-L-Ala-D-Glu-AP-BTH); Column: YMC-Triart C18, 250x4.6 mmL.D. S-5µm, 12nm; gradient: 26% AcCN (0.08% TFA): 74% water (0.08% TFA) isocratic mixture; flow rate: 1 mL/min; detection at 320 nm.

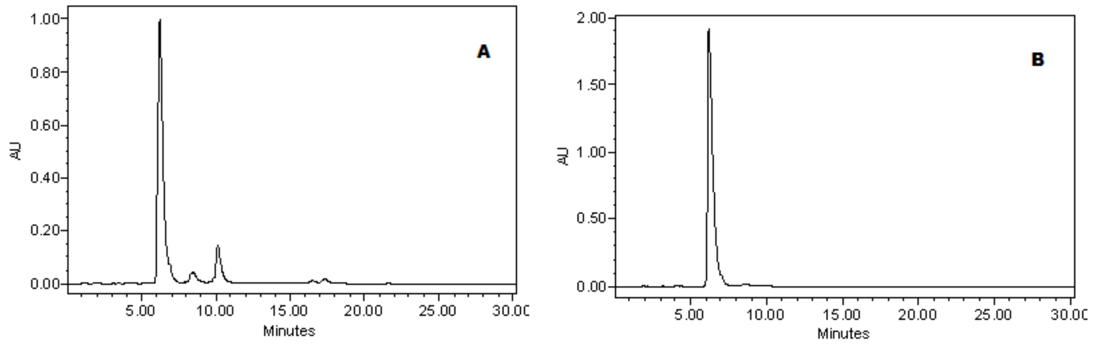

Figure S32. HPLC analysis of **36** (H-L-Ala-L-Lys-AP-BTH) (synthesized by using DIC as the activating agent) after cleavage and treatment using method 1.3.4.3-a (A) and method 1.3.4.3-b (B); Column: Lichrospher RP-18, 5μm, 125-4 mm; gradient: 20% to 100% AcCN in water (both containing 0.08% TFA) in 30 min; flow rate: 1 mL/min; detection at 320 nm.

The eluted peaks were collected and subjected to ESI-MS *m/z*: **36:** [M+H$^+$] found: 426.63.

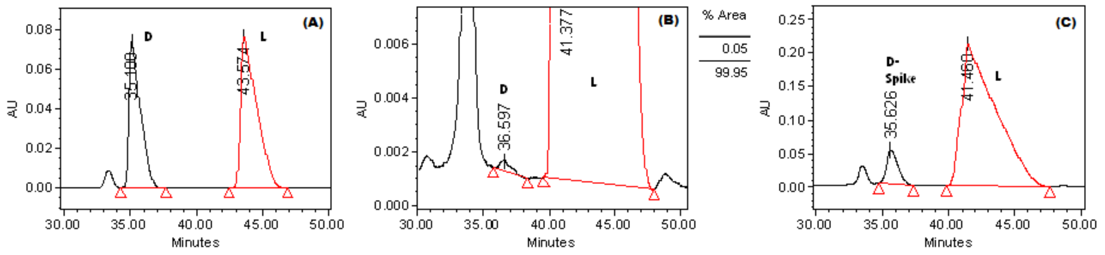

Figure S33. HPLC analysis for determining the enantiomeric purity of the synthesized **36** (H-L-Ala-L-Lys-AP-BTH): (**A**) Mixture of **36** (H-L-Ala-L-Lys-AP-BTH) and its H-L-Ala-D-Lys-AP-BTH diastereomer; (**B**) Crude **36** (synthesized by using DIC as the condensing agent); (**C**) Crude **36** spiked with its D diastereomer (H-L-Ala-D-Lys-AP-BTH); Column: YMC-Triart C18, 250x4.6 mmL.D. S-5µm, 12nm; gradient: 22% AcCN (0.08% TFA): 78% water (0.08% TFA) isocratic mixture; flow rate: 1 mL/min; detection at 320 nm.

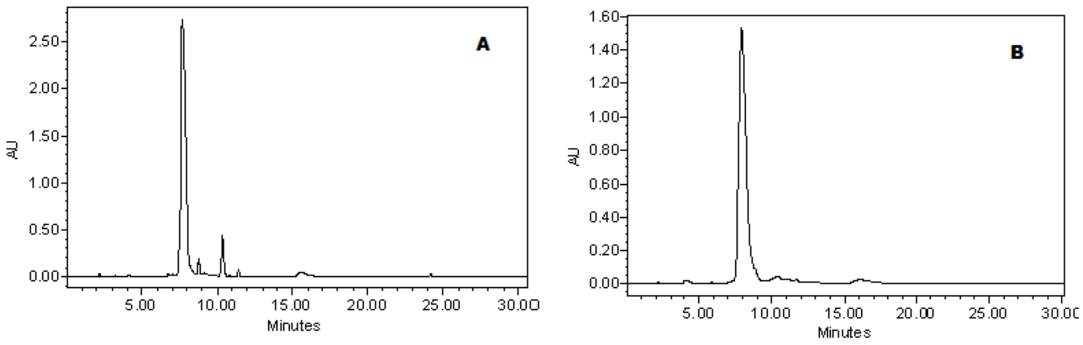

Figure S34. HPLC analysis of **37** (H-L-Lys-L-Ala-L-Cys-AP-BTH) (synthesized by using DIC as the activating agent) after cleavage and treatment using method 1.3.4.3-a (A) and method 1.3.4.3-b (B; Column: Lichrospher RP-18, 5μm, 125-4 mm; gradient: 20% to 100% AcCN in water (both containing 0.08% TFA) in 30 min; flow rate: 1 mL/min; detection at 320 nm.

The eluted peaks were collected and subjected to ESI-MS *m/z*: **37:** [M+H$^+$] found: 529.25.

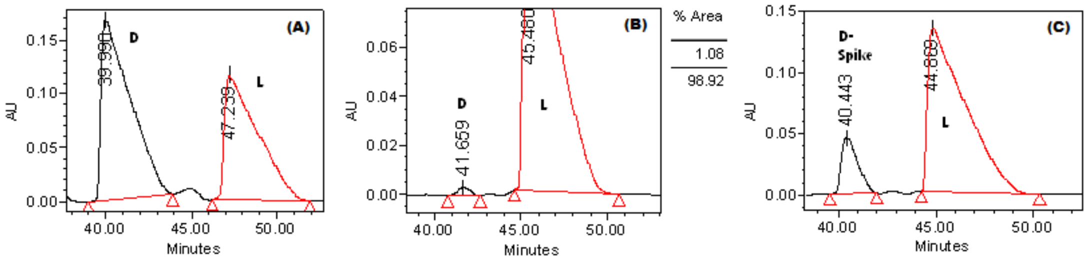

Figure S35. HPLC analysis for determining the enantiomeric purity of the synthesized **37** (H-L-Lys-L-Ala-L-Cys-AP-BTH): (**A**) Mixture of **37** (H-L-Lys-L-Ala-L-Cys-AP-BTH) and its H-L-Lys-L-Ala-D-Cys-AP-BTH diastereomer; (**B**) Crude **37** (synthesized by using DIC as the condensing agent); (**C**) Crude **37** spiked with its D diastereomer (H-L-Lys-L-Ala-D-Cys-AP-BTH); Column: YMC-Triart C18, 250x4.6 mmL.D. S-5µm, 12nm; gradient: 25% AcCN (0.08% TFA): 75% water (0.08% TFA) isocratic mixture; flow rate: 1 mL/min; detection at 320 nm.

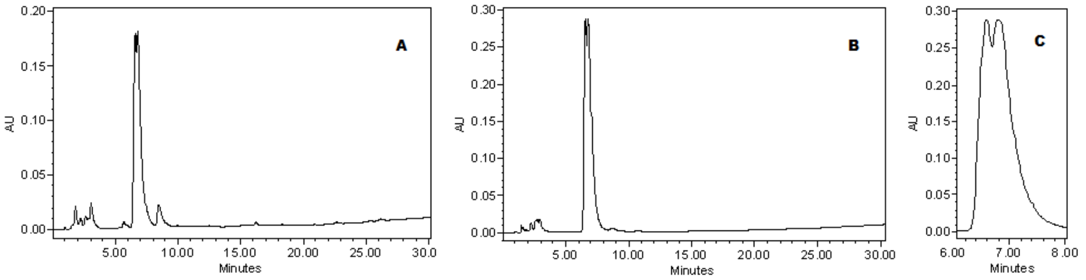

Figure S36. HPLC analysis of **38** (H-L-Ala-L-His-AP-BTH) (synthesized by using DIC as the activating agent) after cleavage and treatment using method 1.3.4.3-a **(A)** and method 1.3.4.3-b **(B)**; **C:** Enlargement of main peak; Column: Lichrospher RP-18, 5μm, 125-4 mm; gradient: 20% to 100% AcCN in water (both containing 0.08% TFA) in 30 min; flow rate: 1 mL/min; detection at 320 nm.

The eluted peaks were collected and subjected to ESI-MS *m/z*: **38:** [M+H$^+$] found: 434.20.

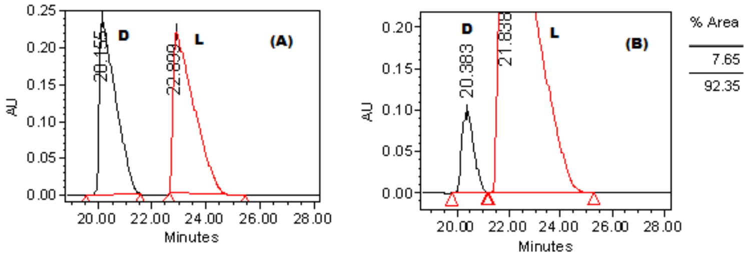

Figure S37. HPLC analysis for determining the enantiomeric purity of the synthesized **38** (H-L-Ala-L-His-AP-BTH): (**A**) Crude **38** (synthesized by using DIC as the condensing agent); (**B**) Crude **38** (synthesized by using HOAt/DIC as the condensing agent); Column: YMC-Triart C18, 250x4.6 mmL.D. S-5µm, 12nm; gradient: 25% AcCN (0.08% TFA): 75% water (0.08% TFA) isocratic mixture; flow rate: 1 mL/min; detection at 320 nm.

F. HPLC/ESI-MS analysis of AP-BTH-peptides (39-43)

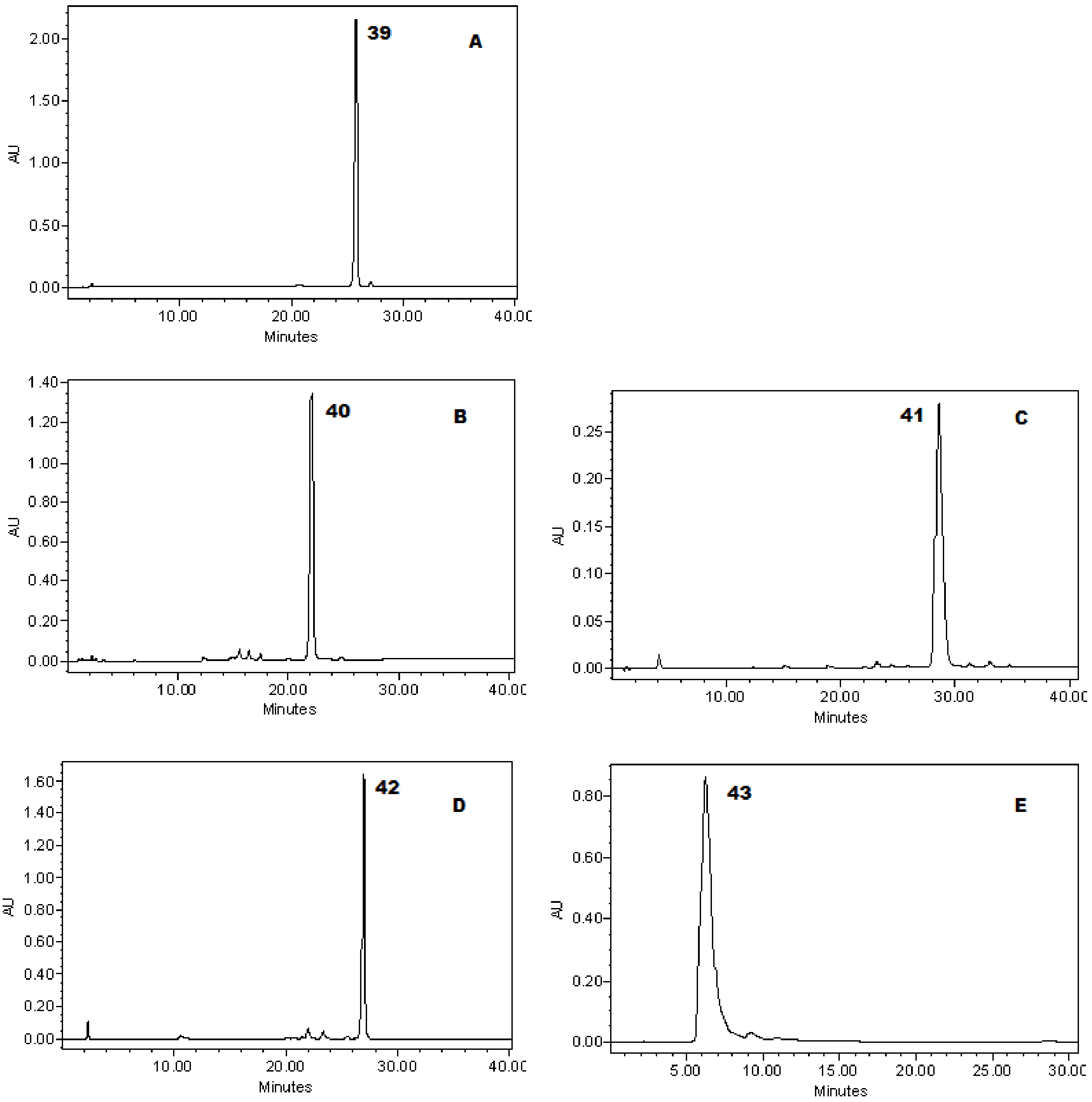

Figure S38. HPLC analysis of **39** (**A**); **40** (**B**); **41** (**C**); **42** (**D**); **43** (**E**); Column: Lichrospher RP-18, 5μm, 125-4 mm: **A: 39** after cleavage and treatment using method 1.3.4.1-b; gradient 20% to 100% AcCN in 30 min; flow rate: 1 mL/min; detection at 320 nm; **B: 40** after cleavage and treatment using method 1.3.4.1-b; gradient 20% to 100% AcCN in 30 min; flow rate: 1 mL/min; detection at 254 nm; **C: 41** after cleavage and treatment using method 1.3.4.3-a; gradient 10% to 60% AcCN in 30 min; flow rate: 1 mL/min; detection at 254 nm; ; **D: 42** after cleavage and treatment using method 1.3.4.1-b; gradient 20% to 100% AcCN in 30 min; flow rate: 1 mL/min; detection at 320 nm; **E: 43** after cleavage and treatment using method 1.3.4.3-a; gradient 20% to 60% AcCN in 30 min; flow rate: 1 mL/min; detection at 320 nm.

The eluted peaks were collected and subjected to ESI-MS *m/z*: **39**: [M+H$^+$] found: 862.52; **40**: [M+H$^+$] found: 819.23; **41**: [M+H$^+$] found: 497.19; **42**: [M+H$^+$] found: 1133.49; **43**: [M+H$^+$] found: 611.52.
